# Exploring the Anti-Pulmonary Fibrosis Mechanism of Jingyin Granule by Network Pharmacology Strategy

**DOI:** 10.3389/fphar.2022.825667

**Published:** 2022-02-11

**Authors:** De-wei Zhu, Qun Yu, Mei-fang Jiang, Dan-dan Wang, Yun-hui Shen

**Affiliations:** ^1^ School of Pharmacy, Shanghai University of Traditional Chinese Medicine, Shanghai, China; ^2^ SPH Xing Ling Sci. & Tech. Pharmaceutical Co., Ltd., Shanghai, China

**Keywords:** pulmonary fibrosis, Jingyin granule, molecular mechanism, UPLC-MS, network pharmacology, signaling pathway analysis

## Abstract

Pulmonary fibrosis (PF) is a clinically common disease caused by many factors, which will lead to lung function decline and even respiratory failure. Jingyin granule has been confirmed to have anti-inflammatory and antiviral effects by former studies, and has been recommended for combating H1N1 influenza A virus (H1N1) infection and Coronavirus disease 2019 (COVID-19) in China. At present, studies have shown that patients with severe COVID-19 infection developed lung fibrotic lesions. Although Jingyin granule can improve symptoms in COVID-19 patients, no study has yet reported whether it can attenuate the process of PF. Here, we explored the underlying mechanism of Jingyin granule against PF by network pharmacology combined with *in vitro* experimental validation. In the present study, the active ingredients as well as the corresponding action targets of Jingyin granule were firstly collected by TCMSP and literature data, and the disease target genes of PF were retrieved by disease database. Then, the common targets were subjected to Gene Ontology (GO) and Kyoto Encyclopedia of Genes and Genomes (KEGG) enrichment analyses, and then a PPI network and an ingredient–target network were constructed. Next, UPLC-MS was used to isolate and identify selected representative components in Jingyin granule. Finally, LPS was used to induce the A549 cell fibrosis model to verify the anti-PF effect of Jingyin granule *in vitro*. Our results indicated that STAT3, JUN, RELA, MAPK3, TNF, MAPK1, IL-6, and AKT1 were core targets of action and bound with good affinity to selected components, and Jingyin granule may alleviate PF progression by Janus kinase 2/signal transducers and activators of transcription (JAK2/STAT3), the mammalian nuclear factor-κB (NF-κB), the phosphatidylinositol 3-kinase (PI3K)/protein kinase B (Akt), tumor necrosis factor (TNF), and the extracellular signal-regulated kinases 1 and 2 (ERK1/2) signaling pathways. Overall, these results provide future therapeutic strategies into the mechanism study of Jingyin granule on PF.

## Highlights

1. Jingyin granule has a potential anti-pulmonary fibrosis effect.
2. The representative active components of Jingyin granule may include arctigenin, quercetin, luteolin, kaempferol, rutin, gallic acid, and chlorogenic acid.
3. The UPLC-MS method was used to identify arctigenin, quercetin, luteolin, kaempferol, rutin, gallic acid, and chlorogenic acid in Jingyin granule.
4. Jingyin granule inhibited the expression of AKT1, JAK2, MAPK1, MAPK3, RELA, PI3K, STAT3, TNF, etc.
5. Jingyin granule may mediate pulmonary fibrosis through JAK2/STAT3, NF-κB, PI3K-AKT, TNF, and ERK1/2 signal pathways.

## Introduction

Fibrosis, which may occur in any organ, is the outcome of dysregulated tissue repair responses to multiple types of tissue injury, particularly during chronic inflammatory disease processes. Pulmonary fibrosis (PF) is an excessive reparative response to tissue injury characterized by spontaneous, progressive scarring of the lungs in the absence of infectious or autoimmune etiologies (Cui et al., 2020). During the development of PF, fibroblast proliferation and extracellular matrix massive aggregation were accompanied by epithelial cell inflammation injury, and the injury site was gradually replaced by fibrous connective tissue to form fibrotic foci. As a fatal malignant disease of the lung, idiopathic pulmonary fibrosis (IPF) has an unknown etiology, a very poor prognosis, and a very high mortality rate, even worse than several cancers, and lung transplantation is the only curative treatment (Vancheri et al., 2010). Europe and North America have a higher incidence with 3–9 new cases per 100,000 per year, whereas Asia and South America report a lower incidence with approximately half a million new cases per year in China (Hutchinson et al., 2015). The incidence of IPF is high in the elderly, and the condition gradually deteriorates with age, and importantly, the survival rate varies greatly among different patients (Kim et al., 2015), with a median survival of 3–5 years following diagnosis (Raghu et al., 2018), and a 3-year survival rate of only 50% (Cui et al., 2020).

Halting IPF progression and curing remain a challenge, though some drugs are able to produce a significant reduction in lung function decline. Based on safety and efficacy in clinical trials, pirfenidone and nintedanib were approved for the treatment of IPF by the Food and Drug Administration (FDA) in 2014, a revolutionary act in conditionally recommending treatment in the 2015 ATS/ERS/JRS/ALAT guideline (Saito et al., 2019). However, the current drugs do not reverse the progression of fibrosis and are associated with side effects such as gastrointestinal intolerance and skin reactions (Guo et al., 2019). Currently, lung transplantation is the only available effective treatment strategy of IPF, but is subject to a limited donor organ supply and the wide variability in clinical course (Collard et al., 2016). Thus, the therapy emphasizes the urgent need to develop novel strategies for the prevention and more effective treatment of this refractory respiratory disease.

Since patients with PF mainly present with progressive dyspnea, cough spitting saliva, chest pain, vomiting, dry mouth, shortness of breath, etc*.*, traditional Chinese medicine (TCM) has summarized it as “lung impediment” or “lung wilting” and the earliest descriptions date back to *The Yellow Emperor’s Inner Canon (first century C)*, the earliest Chinese medical book. It has been generally accepted that PF is mainly caused by the following key pathogenic factors: six-excess external contraction (Wai gan liu yin), internal damage by the seven affects (Nei shang qi qing), qi deficiency (Qi xu), phlegm-stasis (Tan yu), and blood-stasis (Xue yu). Based on pattern identification as the basis for treatment determination, combined with the application of invigorating the blood circulation and transforming phlegm (Huo xue hua tan), supporting and restoring the normal function (Fu zheng gu ben), and so on, TCM treatment of IPF has obvious advantages in improving symptoms and delaying progression.

Jingyin granule is modified from ancient formula Yinqiao Powder, mainly composed of nine herbs [*Nepeta cataria* Linn*.* (Jingjie), *Lonicera japonica* Thunb. (Jingyinhua), *Euonymus japonicus* Thunb. (Sijiqing), *Houttuynia cordata* Thunb. (Yuxingcao), *Indigofera tinctoria* Linn. (Daqingye), *Taraxacum mongolicum* Hand. (Pugongying), *Arctium lappa* L. (Niubangzi), *Saposhnikovia divaricata* (Trucz.) Schischk. (Fangfeng), and *Glycyrrhiza uralensis* Fisch. (Gancao)]. Subject to clinical judgment, it has detoxification, analgesic, and anti-inflammatory effects and have been used for pulmonary wind-heat cold, acute bronchitis, and acute pneumonia for more than 40 years. Guidance is provided on the pulmonary syndrome of mild wind-heat (aversion to cold with fever or no fever, red tongue with thin and yellow fur, sore pharynx, cough, scant sputum, etc*.*) of Coronavirus Disease 2019 (COVID-19) patients by Jingyin granule in “COVID-19 Chinese medicine treatment program (second trial edition) in Shanghai.” Jingyin granule has been recommended to the fourth Shanghai’s medical assistance team to Wuhan and with good clinical effect on COVID-19 in Raytheon Hospital, Wuhan, Hubei.

A network pharmacology study by Wang et al. identified that Jingyin granule could protect against COVID-19 through 88 target genes, among which NOS2, ADAM17, CDK4, MAPK14, and MAPK1 were the top GO-BP enrichment analysis genes (Wang et al., 2021). As indicated in the Guideline on Diagnosis and Treatment of COVID-19 (Trial Version 7th) that was officially issued by the National Health Commission of the People’s Republic of China, interstitial fibrosis of the lung might occur in patients with severe COVID-19 (Zhang et al., 2021). Autopsy on patients who died of COVID-19 also showed disrupted alveolar architecture and fibrosis of the pulmonary interstitium (Huang et al., 2020). Therefore, whether Jingyin granule could prevent PF is worth exploring. The underlying mechanisms should be further explored by pharmacological evaluation, and its potential for the prevention and treatment of PF also needs to be evaluated.

Network pharmacology was first proposed by the UK pharmacologist Andrew L. Hopkins in 2007 (Hopkins, 2007), which integrates several disciplines such as systems biology, network biology, computational biology, multi-target pharmacology, and molecular pharmacology. Network pharmacology is widely used in the research of Chinese medicines as some drugs’ potential targets for combating diseases are obtained by utilizing it. Current network pharmacology has made a pivotal contribution to the development of Chinese medicines in the prevention and treatment of PF diseases as well as in the COVID-19 outbreak (Jin et al., 2021).

This study aimed to observe the effects of Jingyin granule on an *in vitro* cell model of PF, determining the protein expression levels of Janus kinase 2/signal transducers and activators of transcription (JAK2/STAT3), the mammalian nuclear factor-κB (NF-κB), the phosphatidylinositol 3-kinase (PI3K)/protein kinase B (Akt), tumor necrosis factor (TNF), and the extracellular signal-regulated kinases 1 and 2 (ERK1/2), and to investigate the anti-fibrotic mechanism in preventing PF.

## Methods

### Main Candidate Active Ingredients and Targets of Jingyin Granule

The major components of Jingyin granule were obtained by public database screening and literature review. “Jingjie,” “Jinyinhua,” “Sijiqing,” “Yuxingcao,” “Daqingye,” “Niubangzi,” “Fangfeng,” and “Gancao” were respectively retrieved in TCMSP (Traditional Chinese Medicine Systems Pharmacology Database and Analysis Platform, http://tcmspw.com/tcmsp.php), and the active ingredients were selected. The active ingredients of *Taraxacum mongolicum* Hand. (Pugongying) were mainly summarized by searching relevant literatures. The chemical information of main active ingredients was traced back to PubChem database (https://pubchem.ncbi.nlm.nih.gov/). The databases retrieved for potential therapeutic targets of Jingyin granule are the following: TCMSP, SEA (https://sea.bkslab.org/), HitPick (http://mips.helmholtz-muenchen.de/hitpick/), Swiss TargetPrediction (http://www.swisstargetprediction.ch/), and STITCH (http://stitch.embl.de/).

### Collection of Pulmonary Fibrosis-Related Targets

The human target genes related to PF were searched from DisGeNET (https://www.disgenet.org/), GeneCards (https://www.genecards.org/), and OMIM (https://www.omim.org/) potential Disease Targets analysis platforms. After data deduplication/integration, intersecting genes were obtained and considered as therapeutic targets relevant to PF. Drug and corresponding target data that have been validated for the treatment of PF were obtained from DrugBank (https://go.drugbank.com/) and TTD (http://db.idrblab.net/ttd/) database.

### Drug-Target-Disease Network Construction

The predicted action targets of the compounds in Jingyin granule and PF-related disease targets were imported into the Venn online tool (http://www.bioinformatics.com.cn/) to obtain the common targets and Venn diagram. Then, the intersection targets were confirmed by UniProt (https://www.uniprot.org), and Cytoscape software was used to construct a lung fibrosis target network of the main component actions of Jingyin granule.

### Construction of Protein–Protein Interaction Network

To evaluate the importance of the intersection targets, these targets were imported into the STRING database (https://string-db.org/) and Cytoscape software to construct and analyze the PPI network of potential PF targets. The core targets in the PPI network were identified.

### Functional Enrichment Analysis of Shared Targets

To explore the biological process of Jingyin granule attenuating PF, the screened 109 targets were subjected to gene ontology (GO) enrichment analysis and Kyoto Encyclopedia of Genes and Genomes (KEGG) pathway enrichment analysis using the clusterProfiler package based on R language with *p* < 0.05, *q* < 0.05 of filtering thresholds.

### Molecular Docking of Bioactive Components

To verify the binding ability of key components with key targets and explore their accurate binding modes, molecular docking simulation is usually used with the PDB database (http://www.rcsb.org/), PyMOL (2.0) software (http://www.pymol.org/2/), and AutoDock Vina software (http://vina.scripps.edu/). We selected representative targets as receptors in the PPI network and used the representative therapeutic ingredients as the ligand of molecular docking.

### Experiment Validation

#### Materials and Methods

##### Chemicals and Instruments

Arctigenin (CAS: 7770-78-7, MW: 372.41, purity ≥98%), quercetin (CAS: 117-39-5, MW: 302.24, purity ≥97%), luteolin (CAS: 491-70-3, MW: 286.24, purity ≥98%), kaempferol (CAS: 520-18-3, MW: 286.24, purity ≥98%), rutin (CAS: 153-18-4, MW: 610.52, purity ≥98%), gallic acid (CAS: 149-91-7, MW: 170.12, purity ≥98%), and chlorogenic acid (CAS: 327-97-9, MW: 354.31, purity ≥98%) were purchased from Shanghai Yuanye Bio-technology Co. Ltd. (Shanghai, China). A Waters Acquity UPLC coupled with a Xevo G2-XS Q-TOF quadrupole mass spectrometer was used (Waters Co., Milford, MA, United States).

##### UPLC Analysis

The Jingyin granule (2 g, lot# 200302) was dissolved with methanol and vortex mixed for 15 s. The solution was in an ultrasonic water for 30 min. Then, the solution was centrifuged at 12,000 rpm for 10 min, and the supernatant was fixed to 25 ml with methanol. Aliquot (1 μl) was injected into UHPLC-MS for analysis. All separations of Jingyin granule were performed using a Waters Acquity UPLC T3-C18 column (100 × 2.1 mm, 1.7 μm, Waters Co., Milford, MA). The flow rate was 0.2 ml/min, and the column temperature was 40°C. The mobile phase was 0.1% formic acid water (phase A) and methanol (phase B) with gradient elution, and the elution program was: 0–4 min, 90%–75%A; 4–8 min, 75%–75%A; 8–13 min, 75%–40%A; 13–18 min, 40%–25%A; 18–20 min, 25%–2%A; 20–21 min, 2%–90%A; 21–23 min, 90%–90%A.

Mass spectrometric analysis was performed by both positive and negative ion modes, sensitivity mode (resolution: 30,000). Capillary voltage: 3.0 kV; sample cone voltage: 40 V; source offset voltage: 80 V; source temperature: 120°C; desolvation temperature: 450°C; cone gas: 50 L/h; desolvation gas: 800 L/h; nebulizer pressure: 6.0 bar; mass number correction range: *m/z* 50‒800; correction solution: 0.5 mM sodium formate solution; flow rate: 10 μl/min; real-time correction lock spray: 1 ng/UL leucine enkephalin solution, *m/z* 556.2771. Data acquisition was performed using MSE, data types were continuous, energy range was 25–35 V, and scanning time was 0.2 s.

##### Cell Culture

A549 cells were purchased from the Cell Bank of Chinese Academy of Sciences and cultured at 37°C in a humidified atmosphere of 5% CO_2_ and 95% air and in sterile DMEM-H supplemented with 10% fetal bovine serum (Gibco, USA), 100 U/ml penicillin, and 100 ng/ml streptomycin. Cells were seeded in 6-well plates and cultured for 24 h at 37°C. When the A549 cells reached 70%–80% confluence, the culture medium of the cells was replaced with DMEM-H supplemented with 1% fetal bovine serum, 100 U/ml penicillin, and 100 ng/ml streptomycin for 12 h. After that, the cell culture medium was replaced with DMEM-H supplemented with 2% fetal bovine serum, and then treated with 10 μg/ml LPS (from *Escherichia coli* 0111: B4, Sigma-Aldrich, St. Louis, MO, USA) and arctigenin, quercetin, luteolin, kaempferol, rutin, gallic acid, and chlorogenic acid alone or in combination for up to 24 h, respectively.

##### Western Blot Analysis

For Western blot analysis, cell lysate was added after the cells were washed three times by PBS. The isolated proteins were quantified and separated on sodium dodecyl sulfate polyacrylamide gel electrophoresis (SDS-PAGE) (10%), transferred onto a PVDF membrane, and blocked with 5% BSA. After blocking, the proteins were incubated with antibodies overnight at 4°C with primary antibody *β*-actin (1:3,000, Affinity, USA), Phospho-AKT (1:1,000, Proteintech, USA), AKT (1:1,000, CST, USA), JAK2 (1:1,000, CST, USA), Phospho-JAK2 (1:1,000, CST, USA), p44/42 MAPK (ERK1/2) (1:1,000, CST, USA), Phospho-p44/42 MAPK (ERK1/2) (1:1,000, CST, USA), STAT3 (1:1,000, CST, USA), Phospho-STAT3 (1:1,000, CST, USA), NF-κB (1:1,000, CST, USA), Phospho-NF-κB (1:1,000, CST, USA), PI3K (1:1,000, CST, USA), Phospho-PI3K (1:1,000, CST, USA), and TNF-*α* (1:1,000, CST, USA), followed by horseradish peroxidase (HRP)-linked anti-rabbit (1:2,000, CST, USA) or anti-mouse (1:2,000, CST, USA). The protein bands were analyzed by Tanon 4600SF (Tiangong Technology Co., Ltd., Shanghai, China) with chemiluminescence substrate.

##### Statistical Analysis

SPSS 25.0 software and GraphPad Prism 8.0.2 software were used for data analysis and processing, and the results were expressed as mean ± standard deviation (SD). One-way analysis of variance (ANOVA) was used for comparisons between groups. Values of *p* < 0.05 were considered statistically significant.

## Results

**FIGURE 1:**
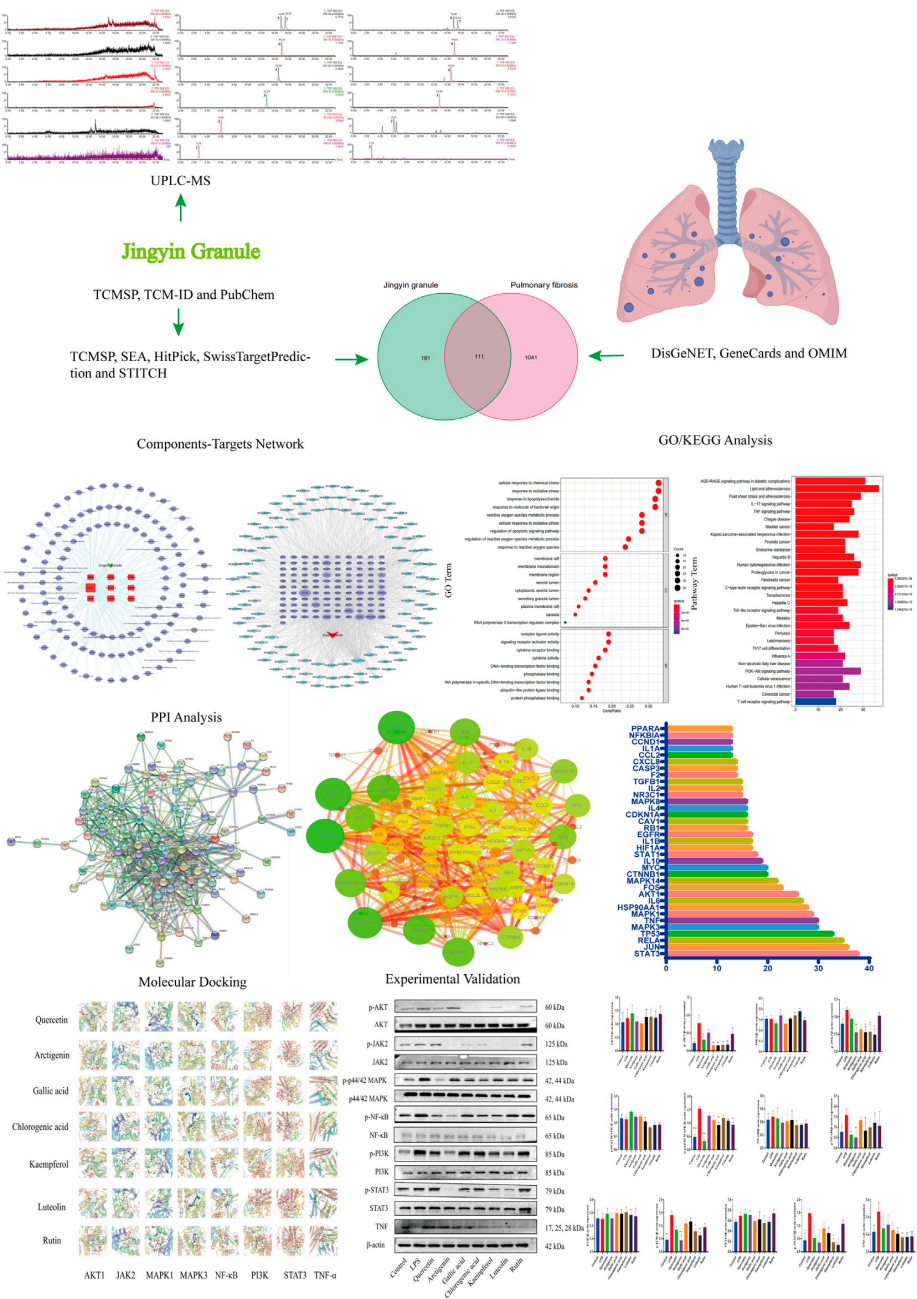
Flow chart of network pharmacology research on the mechanism of Jingyin granule in anti-pulmonary fibrosis.

Based on the multi-component, multi-target, and multi-channel function of Chinese medicines, this study found the mechanism of the anti-PF effect of Jingyin granule, which provides a theoretical basis for Jingyin granule in the treatment of PF. This workflow is shown in Figure 1.

### Active Components and Predicted Therapeutic Targets Results of Jingyin Granule

**FIGURE 2:**
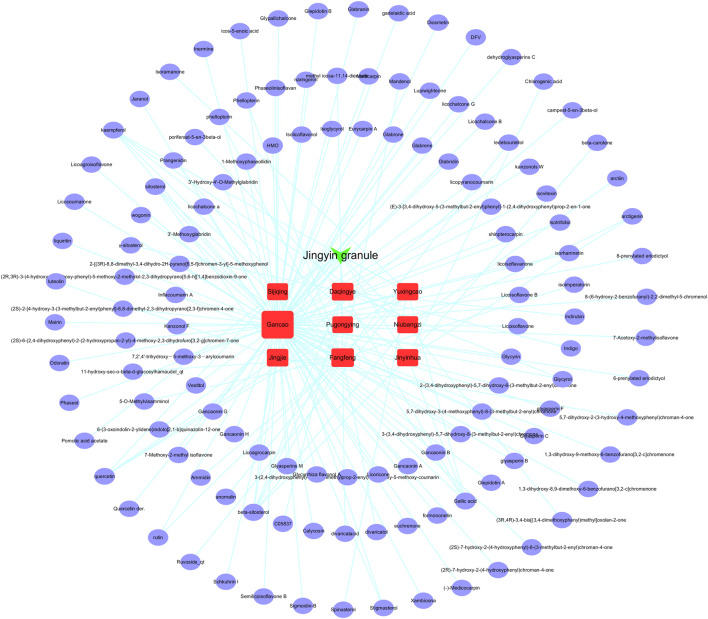
One hundred twenty-six components in Jingyin granule.

A total of 126 major chemical constituents of Jingyin granule were obtained from TCMSP public databases combined with literature screening public databases (Figure 2). ADMET properties of seven of the active ingredients were evaluated using ACD/labs software and the SwissADME online system (http://www.swissadme.ch/), as shown in Table 1. The most relevant targets were selected based on the target information of each database, with TC > 0.4 in SEA, precision >50% in HitPick, the top 15 with the highest scoring value in swisstargetprediction, and stitch based on a score >0.4. A total of 272 potential targets were obtained.

**TABLE 1:** Information on representative chemical constituents of Jingyin granule.

MOL ID	Mol name	CAS	Smile	Formula	Lipinski	Solubility	Bioavailability	GI absorption
MOL000523	Arctigenin	7770-78-7	COC1 = C(C=C(C=C1)CC2COC(=O)C2CC3 = CC(=C(C=C3)O)OC)OC	C_21_H_24_O_6_	Yes	Moderately soluble	0.55	High
MOL000098	Quercetin	117-39-5	C1 = CC(=C(C=C1C2 = C(C(=O)C3 = C(C=C(C=C3O2)O)O)O)O)O	C_15_H_10_O_7_	Yes	Soluble	0.55	High
MOL000006	Luteolin	491-70-3	C1 = CC(=C(C=C1C2 = CC(=O)C3 = C(C=C(C=C3O2)O)O)O)O	C_15_H_10_O_6_	Yes	Soluble	0.55	High
MOL000415	Rutin	153-18-4	CC1C(C(C(C(O1)OCC2C(C(C(C(O2)OC3 = C(OC4 = CC(=CC(=C4C3 = O)O)O)C5 = CC(=C(C=C5)O)O)O)O)O)O)O)O	C_27_H_30_O_16_	No	Soluble	0.17	Low
MOL000513	Gallic acid	149-91-7	C1 = C(C=C(C(=C1O)O)O)C (=O)O	C_7_H_6_O_5_	Yes	Very soluble	0.56	High
MOL000422	Kaempferol	520-18-3	C1 = CC(=CC = C1C2 = C(C(=O)C3 = C(C=C(C=C3O2)O)O)O)O	C_15_H_10_O_6_	Yes	Soluble	0.55	High
MOL003871	Chlorogenic acid	327-97-9	C1C(C(C(CC1(C (=O)O)O)OC(=O)C=CC2 = CC(=C(C=C2)O)O)O)O	C_16_H_18_O_9_	Yes	Very soluble	0.11	Low

### Collection of Disease Targets

**FIGURE 3:**
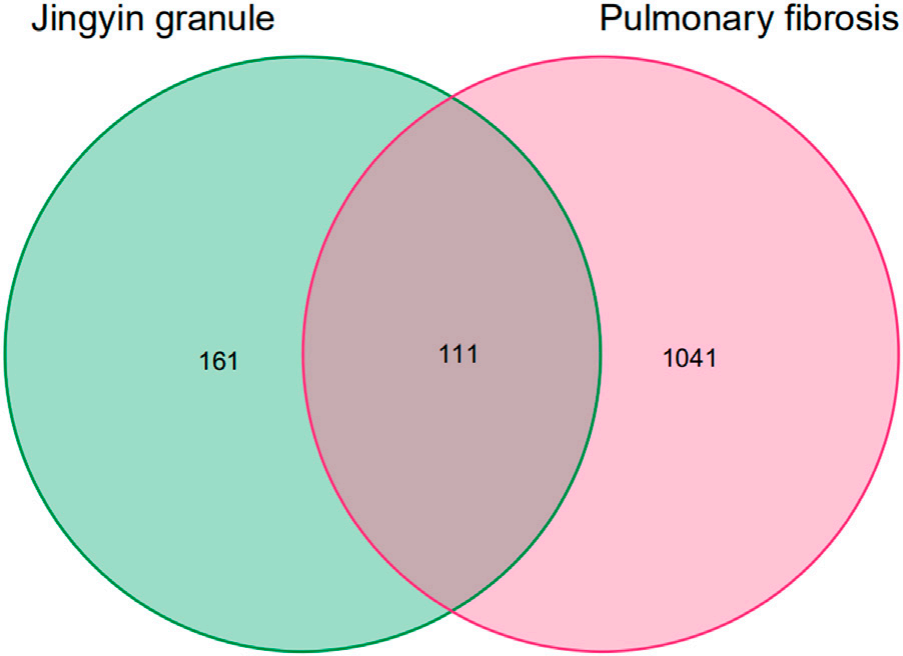
Venn diagram of common targets of pulmonary fibrosis and Jingyin granule.

**FIGURE 4:**
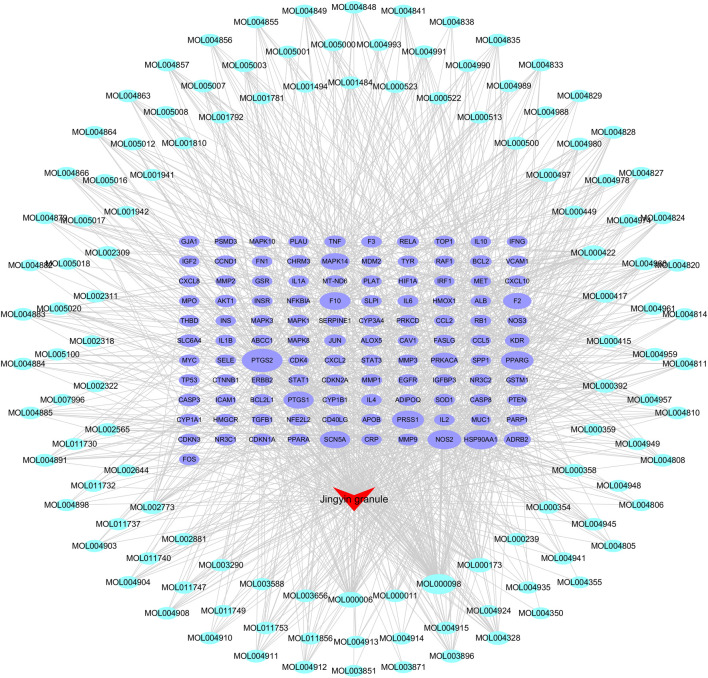
Components–targets network of Jingyin granule against pulmonary fibrosis.

PF-related targets were searched in DisGeNET, GeneCards, OMIM, DrugBank, and TTD databases. A total of 1,152 targets were obtained after merging and deleting duplicate values. As shown in Figure 3, 111 predicted targets were obtained after the Jingyin granule-related targets were mapped to PF-related targets using the Venn. The details of common target genes are shown in Table 2. The 111 targets of intersection were confirmed by UniProt database, and a lung fibrosis–target network of the main components acting on Jingyin granule was constructed by Cytoscape software (Figure 4).

**TABLE 2:** Shared hub targets between pulmonary fibrosis and Jingyin granule.

Number	Gene name	Number	Gene name	Number	Gene name
1	TGFB1	38	CDK4	75	PLAT
2	TNF	39	ALOX5	76	PARP1
3	SLC6A4	40	VCAM1	77	NOS3
4	SCN5A	41	STAT3	78	NFKBIA
5	RELA	42	STAT1	79	NFE2L2
6	PTGS2	43	SOD1	80	MYC
7	PTGS1	44	SLPI	81	MPO
8	PRSS1	45	SELE	82	MMP9
9	PRKACA	46	RB1	83	MMP3
10	PPARG	47	PSMD3	84	MMP2
11	NOS2	48	PPARA	85	MET
12	MAPK14	49	MT-ND6	86	MDM2
13	KDR	50	MAPK8	87	IRF1
14	JUN	51	MAPK3	88	IL2
15	IFNG	52	MAPK10	89	IL1B
16	HSP90AA1	53	MAPK1	90	IL1A
17	F2	54	INSR	91	IL10
18	CYP1A1	55	IL4	92	IGFBP3
19	CHRM3	56	ICAM1	93	IGF2
20	CDKN3	57	HMOX1	94	HIF1A
21	CCL5	58	HMGCR	95	GJA1
22	CASP8	59	GSTM1	96	FOS
23	CASP3	60	GSR	97	F3
24	BCL2	61	CYP3A4	98	ERBB2
25	ADRB2	62	CYP1B1	99	EGFR
26	CCL2	63	APOB	100	CXCL2
27	TP53	64	ADIPOQ	101	CXCL10
28	PRKCD	65	ABCC1	102	CRP
29	NR3C2	66	PTEN	103	CD40LG
30	MMP1	67	CDKN2A	104	CAV1
31	IL6	68	TYR	105	BCL2L1
32	FN1	69	TOP1	106	ALB
33	F10	70	THBD	107	CTNNB1
34	CXCL8	71	SPP1	108	MUC1
35	CDKN1A	72	SERPINE1	109	NR3C1
36	CCND1	73	RAF1	110	INS
37	AKT1	74	PLAU	111	FASLG

### PPI Network Analysis Results

**FIGURE 5:**
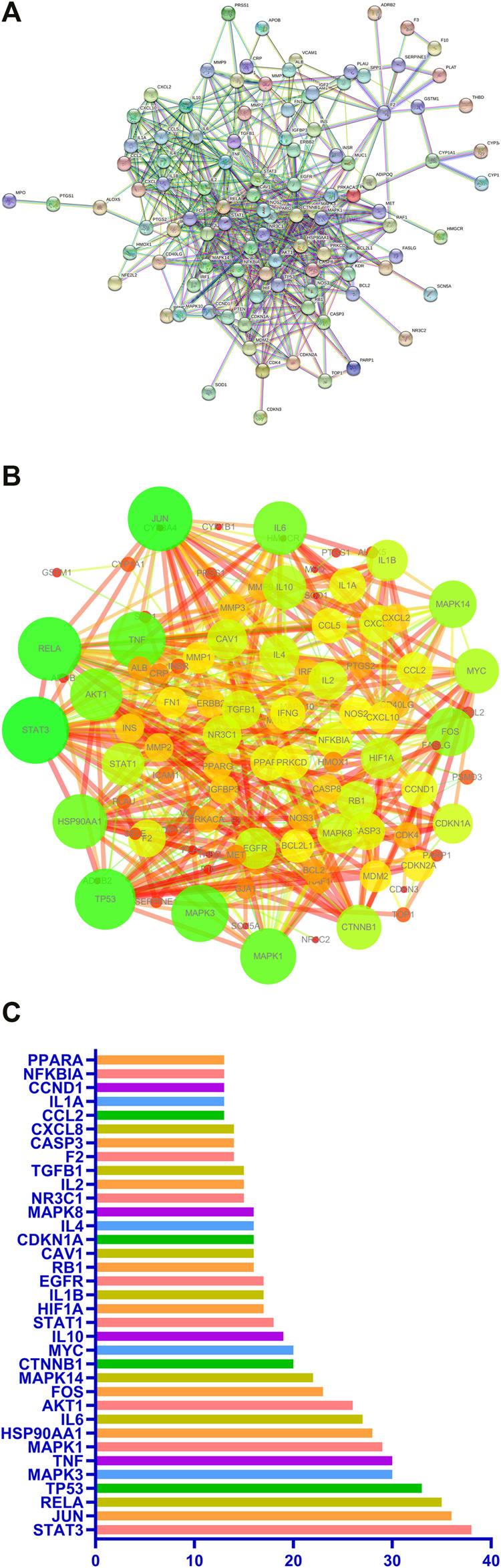
PPI network of potential pulmonary fibrosis targets acted by major components of Jingyin granule **(A,B)**. The top 35 genes in the PPI network **(C)**.

A predicted PPI relationship network about 111 predicted targets was constructed in STRING and visualized by Cytoscape (Figures 5A,B). The 35 genes in the PPI network have much higher network degree value, betweenness, and closeness centrality compared with the other genes (Figure 5C).

### GO Analysis and KEGG Pathway Enrichment Analysis of Shared Targets

**FIGURE 6:**
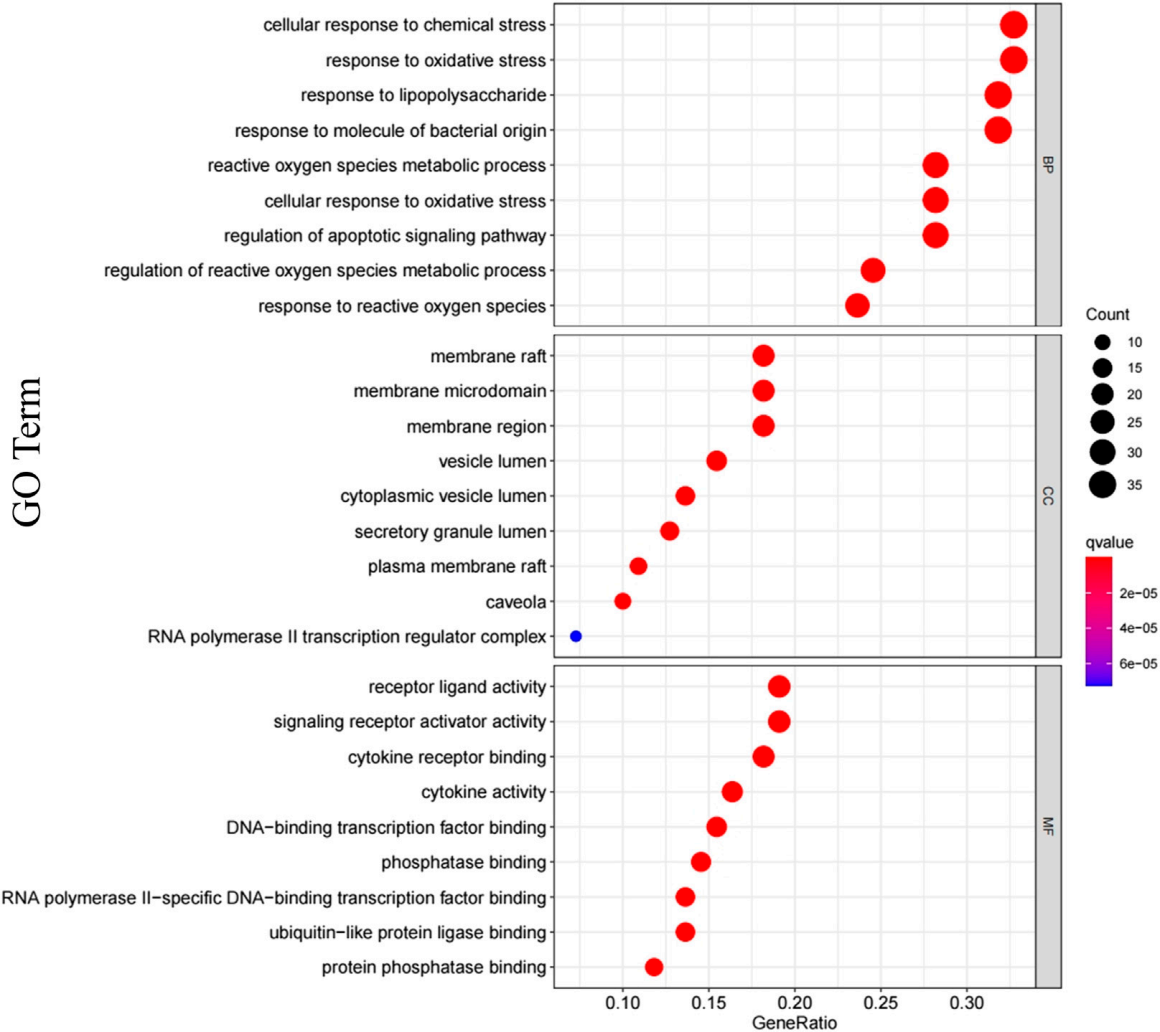
GO functional analysis of the shared targets of Jingyin granule and pulmonary fibrosis.

**FIGURE 7:**
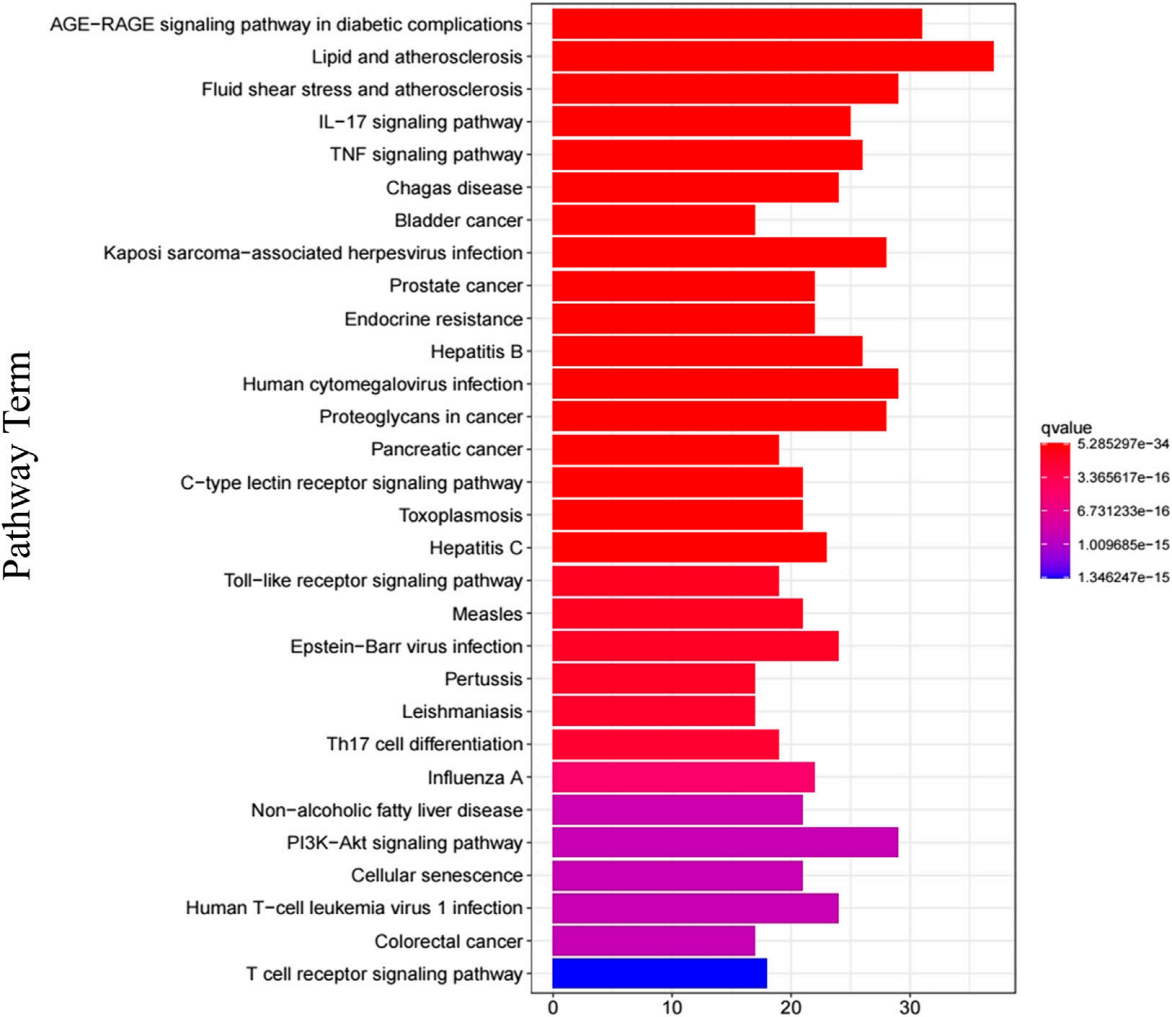
KEGG functional analysis of the shared targets of Jingyin granule and pulmonary fibrosis.

**FIGURE 8:**
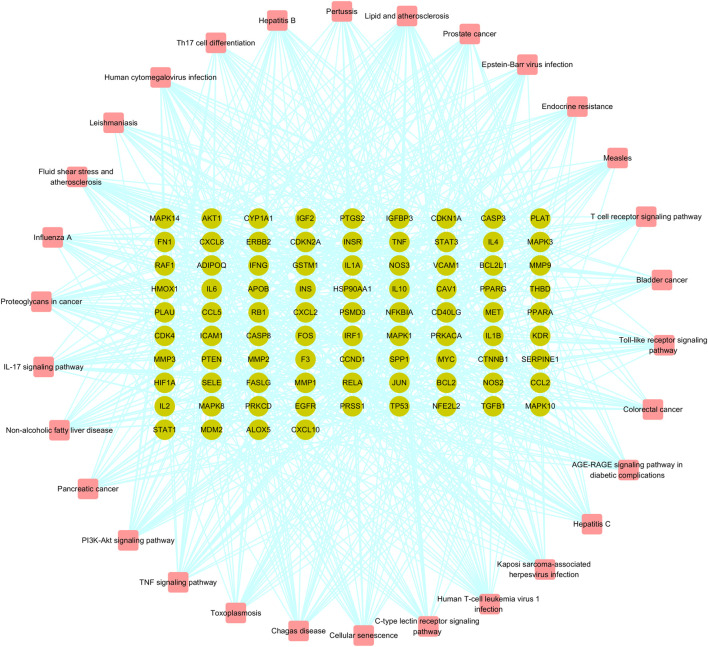
Target genes-pathways network of Jingyin granule.

In order to clarify the biological mechanisms of Jingyin granule against PF, the enrichment analysis of GO and KEGG pathway on 111 targets were performed by the cluster Profiler package based on R language. The GO enrichment analysis annotated the function of key genes from three terms: biological processes (BP), cellular component (CC), and molecular function (MF). A total of 2,709 GO terms were obtained based on *p*-value, and the top 9 items of three parts were selected (Figure 6). The BP was related to cellular response to chemical stress (GO: 0062197), response to oxidative stress (GO: 0006979), response to lipopolysaccharide (GO: 0032496), response to molecule of bacterial origin (GO: 0002237), reactive oxygen species metabolic process (GO: 0072593), cellular response to oxidative stress (GO: 0034599), regulation of apoptotic signaling pathway (GO: 2001233), regulation of reactive oxygen species metabolic process (GO: 2000377), and response to reactive oxygen species (GO: 0000302). The MF was related to receptor ligand activity (GO: 0048018), signaling receptor activator activity (GO: 0030546), cytokine receptor binding (GO: 0005126), cytokine activity (GO: 0005125), DNA-binding transcription factor binding (GO: 0140297), phosphatase binding (GO: 0019902), RNA polymerase II-specific DNA-binding transcription factor binding (GO: 0061629), ubiquitin-like protein ligase binding (GO: 0044389), and protein phosphatase binding (GO: 0019903). The CC was related to membrane raft (GO: 0045121), membrane microdomain (GO: 0098857), membrane region (GO: 0098589), vesicle lumen (GO: 0031983), cytoplasmic vesicle lumen (GO: 0060205), secretory granule lumen (GO: 0034774), plasma membrane raft (GO: 0044853), caveola (GO: 0005901), and RNA polymerase II transcription regulator complex (GO: 0090575). A total of 177 KEGG pathways were obtained based on *p*-value, and the top 30 were selected, such as the AGE-RAGE signaling pathway (hsa04933), TNF signaling pathway (hsa04668), Toll-like receptor (TLR) signaling pathway (hsa04620), and PI3K-Akt signaling pathway (hsa04151) (Figure 7). Then, a Target Genes–Pathways Network was constructed to reveal the relationship between hub targets and pathways intuitively (Figure 8).

### The Molecular Docking of Representative Components and Core Target Proteins

**FIGURE 9:**
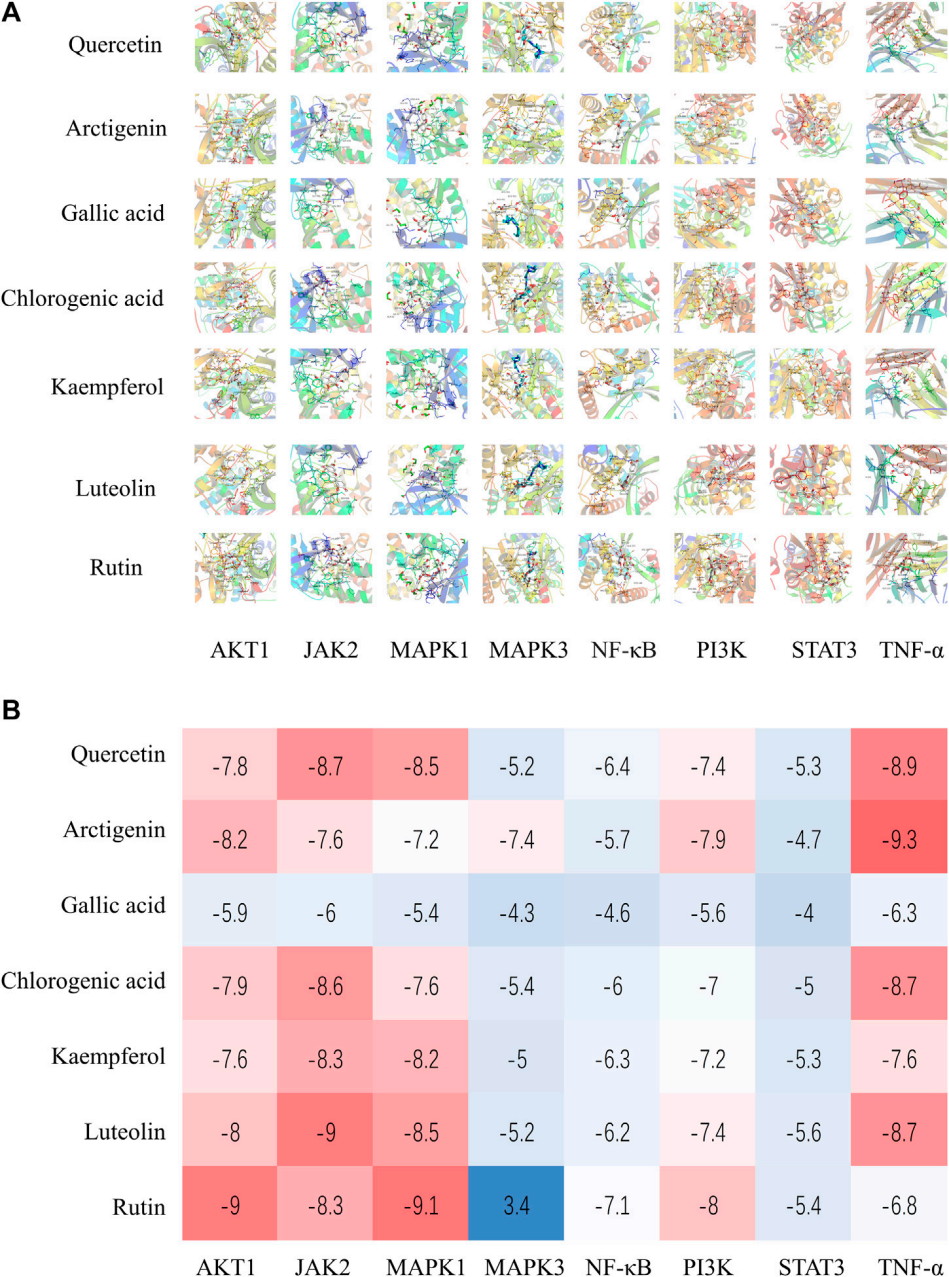
**(A)** Molecular docking results of Jingyin granule components and selected targets. **(B)** The docking scores of Jingyin granule components and core targets.

Seven components were comprehensively selected on the basis of higher degree value in active ingredient–target network, higher content of components in lung, and higher content in Jingyin granule, respectively: arctigenin, quercetin, luteolin, kaempferol, rutin, gallic acid, and chlorogenic acid. The eight core target proteins (AKT1, JAK2, MAPK1, MAPK3, RELA, PI3K, STAT3, and TNF) with high degrees were selected as receptors. The selected compounds showed basically moderate binding potential to receptor proteins with good drug reference values (Figure 9A). The docking scores among them are shown in a heatmap (Figure 9B).

### UPLC-MS Analysis

**FIGURE 10:**
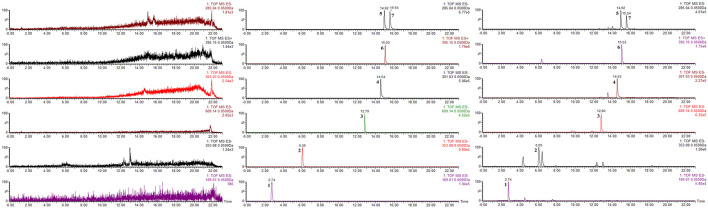
Chromatograms of the seven compounds detected in Jingyin granule. (1: gallic acid, 2: chlorogenic acid, 3: rutin, 4: quercetin, 5: luteolin, 6: arctigenin, 7: kaempferol).

As a rapid, intelligent, reliable, and accurate technique for identification of chemical constituents, UPLC-MS is widely used in the field of TCM. The UPLC-MS method was used to identify the key components previously selected in Jingyin granule, and the typical chromatograms are shown in Figure 10. These seven compounds were identified unambiguously by comparing their accurate masses and retention times with those of pure reference compounds. Molecular weights and details are as shown in Table 3.

**TABLE 3:** Results of qualitative validation for the seven compounds detected in Jingyin granule.

No.	Compound name	CAS	Molecular formula	*m/z*	Retention time
1	Gallic acid	149-91-7	C_7_H_6_O_5_	169.0132	2.74
2	Chlorogenic acid	327-97-9	C_16_H_18_O_9_	353.0854	6.05
3	Rutin	153-18-4	C_27_H_30_O_16_	609.1447	12.79
4	Quercetin	117-39-5	C_15_H_10_O_7_	301.0337	14.54
5	Luteolin	491-70-3	C_15_H_10_O_6_	285.0381	14.92
6	Arctigenin	7770-78-7	C_21_H_24_O_6_	395.1475	15.03
7	Kaempferol	520-18-3	C_15_H_10_O_6_	285.0381	15.54

### Experimental Demonstration *In Vitro*

**FIGURE 11:**
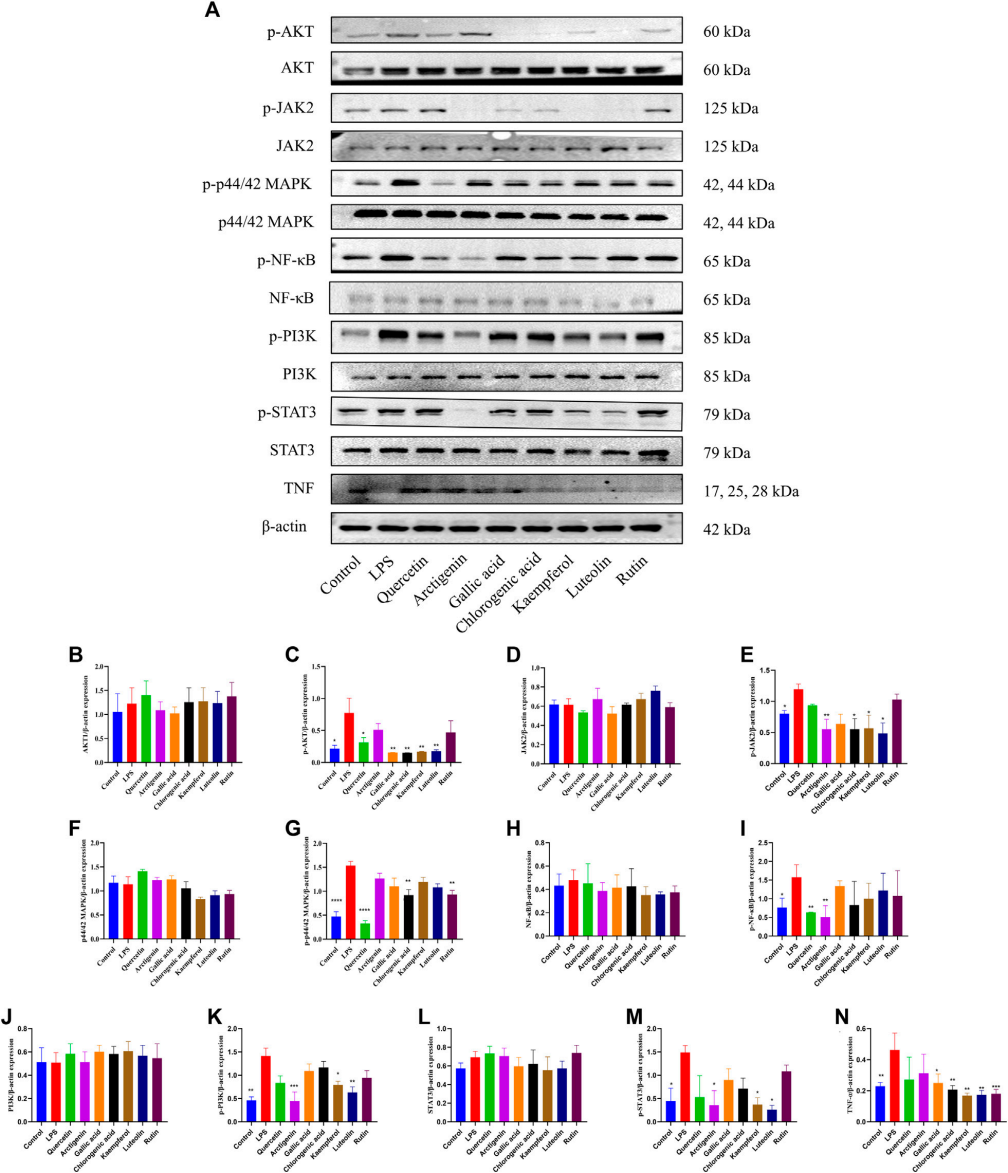
Effects of quercetin, arctigenin, gallic acid, chlorogenic acid, kaempferol, luteolin, and rutin on the protein expression of AKT, p-AKT, JAK2, p-JAK2, p44/42 MAPK (ERK1/2), p-p44/42 MAPK (ERK1/2), NF-κB, p-NF-κB, PI3K, p-PI3K, STAT3, p-STAT3, and TNF-*α* of LPS-induced A549 cells **(A–N)**. *p < 0.05, **p < 0.01, compared with the LPS group.

Based on the results of the PPI network, active compounds–disease targets network, and literature data, seven components (arctigenin, quercetin, luteolin, kaempferol, rutin, gallic acid, and chlorogenic acid) and eight targets (AKT1, JAK2, MAPK1, MAPK3, RELA, PI3K, STAT3, and TNF) played crucial roles in anti-PF. To explore the effects of anti-PF, 50 μM quercetin, 200 μM arctigenin, 75 μM gallic acid, 400 μM chlorogenic acid, 50 μM kaempferol, 50 μM luteolin, and 40 μM rutin were used. As per the results obtained, the expression of AKT1, JAK2, MAPK1, MAPK3, RELA, PI3K, STAT3, and TNF was decreased significantly after drug intervention compared with that in the model group (*p* < 0.05 or *p* < 0.01) (Figures 11A–N).

## Discussion

The lung has the remarkable ability to repair and recover in response to constant exposure to many injuries, through a cascade of finally synchronized biological processes (Martinez et al., 2017). However, repeated microinjury to alveolar epithelial tissues may result in loss of epithelial integrity and dysregulation of regeneration; excessive repair may predispose the individual to PF. Those external stressors of epithelial damage include environmental and occupational factors such as smoking, viral infections and peripheral lung traction injury, genetic risk, and certain comorbidities (Selman et al., 2001). IPF, as a specific form of chronic, progressive lung disease of unknown cause, is characterized by decline in lung function, worsening quality of life, and early mortality (Cottin et al., 2019), but all currently relevant drugs can only delay lung failure, not reverse the course of PF, and have unavoidable side effects.

Based on syndrome differentiation, IPF is treated through a staged, multi-level dialectical application, and more comprehensive ideas and methods are also being used by TCM, such as staging treatment, prescription treatment, collateral treatment, and acupuncture combined with internal and external treatment (Zhang et al., 2021). Either as monotherapy or in combination with standard Western medical treatment, Chinese medicines usually exert a wider action spectrum in managing the entire medical disorders by the effects of synergism and attenuation (Wang, 2013). Multiple studies have demonstrated that the active agents of single herbs and Chinese medicine formulas, in particular, the flavonoids, terpenes, and alkaloids, have significant therapeutic effects on IPF, the related mechanisms of which appear to involve the regulation of inflammation, oxidant stress, and pro-fibrotic signaling pathways, among others (Li and Kan, 2017).

Jingyin granule obtained new drug approval in China in 2009 as a prescription for anti-atypical pneumonia and is recommended as a reserve medication in Shanghai for the prevention and treatment of H1N1 infection and COVID-19. The prescription of Jingyin granule is complex with many ingredients, some of which have been proven to possess potential benefits in antifibrotic treatment. Miao et al. (2019) demonstrated the protective effects of the water extract of *Lonicera japonica* Thunb. from liver fibrosis in mice treated with carbon tetrachloride (CCl_4_), the mechanism of which was inhibition of hepatic stellate cells (HSCs) activation, liver oxidative stress injury, and the epithelial–mesenchymal transition (EMT) process. The current study found that *H. cordata* Thunb. aqueous extract had obvious antioxidant and protective effects on rats with bleomycin-induced PF (Ng et al., 2007). Our previous network pharmacology study of *H. cordata* Thunb. identified that it may act through multiple signaling pathways to alleviate PF (Zhu et al., 2021). Our *in vivo* experimental study also clarified that sodium houttuyfonate, an adduct compound of houttuynin and sodium bisulfite, may alleviate the degree of fibrosis in bleomycin-induced PF model mice through the transforming growth factor-*β* (TGF-*β*)/Smads pathway (Shen et al., 2021). Arctigenin, the main element of *A. lappa* L., has multiple anti-visceral fibrotic functions, such as reversing TGF-*β*1-induced renal tubular EMT-like changes (Li et al., 2015) and reversing the EMT process in alveolar type II cells in paraquat (PQ)-induced lung fibrosis by the Wnt3a/*β*-catenin pathway (Gao et al., 2020). In addition, glycyrrhizic acid has been reported to show antifibrosis outcomes in PF and liver fibrosis (Gao et al., 2015; Liang et al., 2015).

We screened out a total of 126 active compounds (after deduplication) from 9 kinds of medicinal herbs in Jingyin granule through TCMSP database and literature search, according to the ADME principle (setting OB ≥ 30 and DL ≥ 0.18) (Tsaioun et al., 2016). Their corresponding target genes were then retrieved through the database and intersected on PF-related disease targets, resulting in 111 common target genes. The KEGG pathway enrichment analysis revealed the 142 significant (*p* < 0.001) possible pathways against PF including AGE-RAGE, TNF, IL-17, TLR, PI3K/Akt, and MAPK signaling pathway. After that, a PPI predictive relationship network of 111 predicted targets was constructed and revealed the top 10 hub genes, namely, STAT3, JUN, RELA, TP53, MAPK3, TNF, MAPK1, HSP90AA1, IL6, and AKT1.

IL-6 is a proinflammatory factor that was found to be elevated in the serum of patients with fibrotic diseases (Le et al., 2014). STATs mainly function as transcription factors and have seven member families (STAT1, STAT2, STAT3, STAT4, STAT5A, STAT5b, and STAT6) that regulate several physiological life activities, such as anti-inflammation. STAT3 together with JAK2 and their phosphorylated forms have all been examined in fibrotic lungs from patients with IPF (Milara et al., 2018). The IL-6/STAT3 signaling axis has been shown to play essential roles in the development of inflammatory and fibrotic diseases (O'Donoghue et al., 2012). JAKs are triggered by the activation of members of the IL-6 family that mediates subsequent phosphorylation and activation of STAT3. IL-6-mediated JAK/STAT3 signaling plays a vital role in the airway remodeling of asthma, and its inhibition prevents airway inflammation and remodeling, and blocks Th2 and Th17 cell expansion in a murine asthma model (Gavino et al., 2016). JUN is a component of the AP1 family of transcription factors that coordinates the transcriptional regulation of a multitude of genes that are essential for many cellular processes, including differentiation, proliferation, and apoptosis. JUN is highly expressed in all major human fibrotic conditions and as a downstream gene of MAPK-signaling cascades (Wernig et al., 2017). It has been proved that Jun-mediated CD47 enhancer activation can be amplified by IL-6, resulting in increased CD47 protein expression and induction of profibrotic and immunosuppressive gene expression (Cui et al., 2020). P65-RelA is a subunit of the NF-κB family. NF-κB activation is generally considered central to the innate immune response and will lead to exacerbated chronic inflammation and further reduced lung function. MAPK3 (ERK1) and MAPK1 (ERK2), the earliest identified MAPK pathways, are involved in growth factor signaling and regulate various cellular processes including cell proliferation, differentiation, and apoptosis. ERK1/2 can be activated by the IL-6-type family cytokine (Heinrich et al., 2003). Galuppo et al. (2011) confirmed that inhibition of MEK and ERK1/2 by PD98059, a highly selective inhibitor of MAP/ERK kinase1 (MEK1) activation, reduces lung injury and inflammation in a mouse model of bleomycin-induced PF. Similarly, Madala et al. (2012) demonstrated that selective inhibition of MEK prevents progression of lung fibrosis in the TGF-*α*-induced model. However, current studies also found that ERK1/2 activation in epithelial and endothelial cells subsides, accompanying progression of fibrosis in IPF, and by the later stage of IPF when fibroblast differentiation predominates, inhibition of ERK1/2 may promote IPF progression instead (Yoshida et al., 2002; Lai et al., 2016). AKT1, also referred to as protein kinase B alpha, is one of the three members of the human AKT serine–threonine protein kinase family. It has been shown that p-AKT1 was increased 3-fold in alveolar macrophages of IPF patients compared with normal tissues, and an increase in p-AKT1 expression was also observed in macrophages isolated from the lungs of BLM injured mice (Larson-Casey et al., 2016). Overexpressed AKT1 can increase mitophagy to increase macrophage-derived TGF-*β*1 expression and apoptosis resistance, thereby promoting fibrosis progression in mice.

Evidence suggests that lipopolysaccharide (LPS), as one of the pathogens that induce acute lung injury, can increase reactive oxygen species (ROS) and TGF-*β* production and macrophage infiltration, thereby promoting alveolar epithelial mesenchymal transition (EMT) and lung fibrosis (Qiu et al., 2019; Ding et al., 2020).

In this study, arctigenin, quercetin, luteolin, kaempferol, rutin, gallic acid, and chlorogenic acid in Jingyin granule were used for a functional study due to more connected targets in the ingredient–target network and more distribution in lung tissue in a pharmacokinetic study. Firstly, Jingyin granule was analyzed by a simple and accurate HPLC method, UPLC-MS, for the simultaneous separation and identification of the seven ingredients for functional evaluation. Molecular docking indicated that the key components combined well with target proteins. *In vitro* cell experiments proved that the important ingredients, arctigenin and luteolin, inhibited the production of fibrosis in LPS-induced A549 cells by suppressing the JAK2/STAT3, NF-κB, PI3K-AKT, TNF, and ERK1/2 signaling pathways. Moreover, we are doing the *in vivo* experiment to provide more mechanism study for the anti-fibrosis effect of Jingyin granule.

## Conclusion

tIn conclusion, our present study systematically analyzed the related targets and signaling pathways of Jingyin granule against PF and conducted an *in vitro* experiment and demonstrated that Jingyin granule obviously inhibited the activation of JAK2/STAT3, NF-κB, PI3K/AKT, TNF, and ERK1/2 signaling pathways in LPS-induced PF. This underlying mechanism we elucidated may become a theoretical basis for the use of Jingyin granule for the treatment of PF.

## Data Availability

The original contributions presented in the study are included in the article/Supplementary Material, further inquiries can be directed to the corresponding author.

## References

[B1] Collard H. R. Ryerson C. J. Corte T. J. Jenkins G. Kondoh Y. Lederer D. J. (2016). Acute Exacerbation of Idiopathic Pulmonary Fibrosis. An International Working Group Report. Am. J. Respir. Crit. Care Med. 194 (3), 265–275. 10.1164/rccm.201604-0801CI 27299520

[B2] Cottin V. Wollin L. Fischer A. Quaresma M. Stowasser S. Harari S. (2019). Fibrosing Interstitial Lung Diseases: Knowns and Unknowns. Eur. Respir. Rev. 28 (151), 180100. 10.1183/16000617.0100-2018 30814139 PMC9489101

[B3] Cui L. Chen S. Y. Lerbs T. Lee J. W. Domizi P. Gordon S. (2020). Activation of JUN in Fibroblasts Promotes Pro-fibrotic Programme and Modulates Protective Immunity. Nat. Commun. 11 (1), 2795. 10.1038/s41467-020-16466-4 32493933 PMC7270081

[B4] Ding Z. Wu X. Wang Y. Ji S. Zhang W. Kang J. (2020). Melatonin Prevents LPS-Induced Epithelial-Mesenchymal Transition in Human Alveolar Epithelial Cells via the GSK-3β/Nrf2 Pathway. Biomed. Pharmacother. 132, 110827. 10.1016/j.biopha.2020.110827 33065391

[B5] Galuppo M. Esposito E. Mazzon E. Di Paola R. Paterniti I. Impellizzeri D. (2011). MEK Inhibition Suppresses the Development of Lung Fibrosis in the Bleomycin Model. Naunyn Schmiedebergs Arch. Pharmacol. 384 (1), 21–37. 10.1007/s00210-011-0637-7 21533992

[B6] Gao F. Zhang Y. Yang Z. Wang M. Zhou Z. Zhang W. (2020). Arctigenin Suppressed Epithelial-Mesenchymal Transition through Wnt3a/β-Catenin Pathway in PQ-Induced Pulmonary Fibrosis. Front. Pharmacol. 11, 584098. 10.3389/fphar.2020.584098 33390951 PMC7772408

[B7] Gao L. Tang H. He H. Liu J. Mao J. Ji H. (2015). Glycyrrhizic Acid Alleviates Bleomycin-Induced Pulmonary Fibrosis in Rats. Front. Pharmacol. 6, 215. 10.3389/fphar.2015.00215 26483688 PMC4589765

[B8] Gavino A. C. Nahmod K. Bharadwaj U. Makedonas G. Tweardy D. J. (2016). STAT3 Inhibition Prevents Lung Inflammation, Remodeling, and Accumulation of Th2 and Th17 Cells in a Murine Asthma Model. Allergy 71 (12), 1684–1692. 10.1111/all.12937 27225906

[B9] Guo J. Li B. Wu W. Wang Z. Wang F. Guo T. (2019). Chinese Herbal Medicines Compared with N-Acetylcysteine for the Treatment of Idiopathic Pulmonary Fibrosis: A Systematic Review of Randomized Controlled Trials. Evid. Based Complement. Alternat Med. 2019, 5170638. 10.1155/2019/5170638 31312224 PMC6595365

[B10] Heinrich P. C. Behrmann I. Haan S. Hermanns H. M. Müller-Newen G. Schaper F. (2003). Principles of Interleukin (IL)-6-type Cytokine Signalling and its Regulation. Biochem. J. 374 (Pt 1), 1–20. 10.1042/bj20030407 12773095 PMC1223585

[B11] Hopkins A. L. (2007). Network Pharmacology. Nat. Biotechnol. 25 (10), 1110–1111. 10.1038/nbt1007-1110 17921993

[B12] Huang Y. Tan C. Wu J. Chen M. Wang Z. Luo L. (2020). Impact of Coronavirus Disease 2019 on Pulmonary Function in Early Convalescence Phase. Respir. Res. 21 (1), 163. 10.1186/s12931-020-01429-6 32600344 PMC7323373

[B13] Hutchinson J. Fogarty A. Hubbard R. McKeever T. (2015). Global Incidence and Mortality of Idiopathic Pulmonary Fibrosis: a Systematic Review. Eur. Respir. J. 46 (3), 795–806. 10.1183/09031936.00185114 25976683

[B14] Jin D. An X. Zhang Y. Zhao S. Duan L. Duan Y. (2021). Potential Mechanism Prediction of Herbal Medicine for Pulmonary Fibrosis Associated with SARS-CoV-2 Infection Based on Network Analysis and Molecular Docking. Front. Pharmacol. 12, 602218. 10.3389/fphar.2021.602218 33986661 PMC8112227

[B15] Kim H. J. Perlman D. Tomic R. (2015). Natural History of Idiopathic Pulmonary Fibrosis. Respir. Med. 109 (6), 661–670. 10.1016/j.rmed.2015.02.002 25727856

[B16] Lai J. M. Zhang X. Liu F. F. Yang R. Li S. Y. Zhu L. B. (2016). Redox-sensitive MAPK and Notch3 Regulate Fibroblast Differentiation and Activation: a Dual Role of ERK1/2. Oncotarget 7 (28), 43731–43745. 10.18632/oncotarget.9667 27248323 PMC5190056

[B17] Larson-Casey J. L. Deshane J. S. Ryan A. J. Thannickal V. J. Carter A. B. (2016). Macrophage Akt1 Kinase-Mediated Mitophagy Modulates Apoptosis Resistance and Pulmonary Fibrosis. Immunity 44 (3), 582–596. 10.1016/j.immuni.2016.01.001 26921108 PMC4794358

[B18] Le T. T. Karmouty-Quintana H. Melicoff E. Le T. T. Weng T. Chen N. Y. (2014). Blockade of IL-6 Trans Signaling Attenuates Pulmonary Fibrosis. J. Immunol. 193 (7), 3755–3768. 10.4049/jimmunol.1302470 25172494 PMC4169999

[B19] Li A. Wang J. Zhu D. Zhang X. Pan R. Wang R. (2015). Arctigenin Suppresses Transforming Growth Factor-Β1-Induced Expression of Monocyte Chemoattractant Protein-1 and the Subsequent Epithelial-Mesenchymal Transition through Reactive Oxygen Species-dependent ERK/NF-κB Signaling Pathway in Renal Tubular Epithelial Cells. Free Radic. Res. 49 (9), 1095–1113. 10.3109/10715762.2015.1038258 25968940

[B20] Li L. C. Kan L. D. (2017). Traditional Chinese Medicine for Pulmonary Fibrosis Therapy: Progress and Future Prospects. J. Ethnopharmacol 198, 45–63. 10.1016/j.jep.2016.12.042 28038955 PMC7127743

[B21] Liang B. Guo X. L. Jin J. Ma Y. C. Feng Z. Q. (2015). Glycyrrhizic Acid Inhibits Apoptosis and Fibrosis in Carbon-Tetrachloride-Induced Rat Liver Injury. World J. Gastroenterol. 21 (17), 5271–5280. 10.3748/wjg.v21.i17.5271 25954100 PMC4419067

[B22] Madala S. K. Schmidt S. Davidson C. Ikegami M. Wert S. Hardie W. D. (2012). MEK-ERK Pathway Modulation Ameliorates Pulmonary Fibrosis Associated with Epidermal Growth Factor Receptor Activation. Am. J. Respir. Cel Mol Biol 46 (3), 380–388. 10.1165/rcmb.2011-0237OC PMC3326433 22021337

[B23] Martinez F. J. Collard H. R. Pardo A. Raghu G. Richeldi L. Selman M. (2017). Idiopathic Pulmonary Fibrosis. Nat. Rev. Dis. Primers 3, 17074. 10.1038/nrdp.2017.74 29052582

[B24] Miao H. Zhang Y. Huang Z. Lu B. Ji L. (2019). Lonicera japonica Attenuates Carbon Tetrachloride-Induced Liver Fibrosis in Mice: Molecular Mechanisms of Action. Am. J. Chin. Med. 47 (2), 351–367. 10.1142/s0192415x19500174 30871359

[B25] Milara J. Hernandez G. Ballester B. Morell A. Roger I. Montero P. (2018). The JAK2 Pathway Is Activated in Idiopathic Pulmonary Fibrosis. Respir. Res. 19 (1), 24. 10.1186/s12931-018-0728-9 29409529 PMC5801676

[B26] Ng L. T. Yen F. L. Liao C. W. Lin C. C. (2007). Protective Effect of Houttuynia Cordata Extract on Bleomycin-Induced Pulmonary Fibrosis in Rats. Am. J. Chin. Med. 35 (3), 465–475. 10.1142/s0192415x07004989 17597505

[B27] O'Donoghue R. J. Knight D. A. Richards C. D. Prêle C. M. Lau H. L. Jarnicki A. G. (2012). Genetic Partitioning of Interleukin-6 Signalling in Mice Dissociates Stat3 from Smad3-Mediated Lung Fibrosis. EMBO Mol. Med. 4 (9), 939–951. 10.1002/emmm.201100604 22684844 PMC3491826

[B28] Qiu P. Liu Y. Zhang J. (2019). Recent Advances in Studies of Molecular Hydrogen against Sepsis. Int. J. Biol. Sci. 15 (6), 1261–1275. 10.7150/ijbs.30741 31223285 PMC6567800

[B29] Raghu G. Remy-Jardin M. Myers J. L. Richeldi L. Ryerson C. J. Lederer D. J. (2018). Diagnosis of Idiopathic Pulmonary Fibrosis. An Official ATS/ERS/JRS/ALAT Clinical Practice Guideline. Am. J. Respir. Crit. Care Med. 198 (5), e44–e68. 10.1164/rccm.201807-1255ST 30168753

[B30] Saito S. Alkhatib A. Kolls J. K. Kondoh Y. Lasky J. A. (2019). Pharmacotherapy and Adjunctive Treatment for Idiopathic Pulmonary Fibrosis (IPF). J. Thorac. Dis. 11 (Suppl. 14), S1740–s1754. 10.21037/jtd.2019.04.62 31632751 PMC6783717

[B31] Selman M. King T. E. Pardo A. (2001). Idiopathic Pulmonary Fibrosis: Prevailing and Evolving Hypotheses about its Pathogenesis and Implications for Therapy. Ann. Intern. Med. 134 (2), 136–151. 10.7326/0003-4819-134-2-200101160-00015 11177318

[B32] Shen Y. H. Cheng M. H. Liu X. Y. Zhu D. W. Gao J. (2021). Sodium Houttuyfonate Inhibits Bleomycin Induced Pulmonary Fibrosis in Mice. Front. Pharmacol. 12, 596492. 10.3389/fphar.2021.596492 33716736 PMC7947865

[B33] Tsaioun K. Blaauboer B. J. Hartung T. (2016). Evidence-based Absorption, Distribution, Metabolism, Excretion (ADME) and its Interplay with Alternative Toxicity Methods. Altex 33 (4), 343–358. 10.14573/altex.1610101 27806179

[B34] Vancheri C. Failla M. Crimi N. Raghu G. (2010). Idiopathic Pulmonary Fibrosis: a Disease with Similarities and Links to Cancer Biology. Eur. Respir. J. 35 (3), 496–504. 10.1183/09031936.00077309 20190329

[B35] Wang B. Sun X. Kong X. Gao Y. (2021). Systematic Elucidation of the Mechanism of Jingyin Granule in the Treatment of Novel Coronavirus (COVID-19) Pneumonia via Network Pharmacology. Int. J. Med. Sci. 18 (7), 1648–1656. 10.7150/ijms.53575 33746581 PMC7976572

[B36] Wang J. (2013). Treatment of Food Anaphylaxis with Traditional Chinese Herbal Remedies: from Mouse Model to Human Clinical Trials. Curr. Opin. Allergy Clin. Immunol. 13 (4), 386–391. 10.1097/ACI.0b013e3283615bc4 23799334 PMC4276320

[B37] Wernig G. Chen S. Y. Cui L. Van Neste C. Tsai J. M. Kambham N. (2017). Unifying Mechanism for Different Fibrotic Diseases. Proc. Natl. Acad. Sci. U S A. 114 (18), 4757–4762. 10.1073/pnas.1621375114 28424250 PMC5422830

[B38] Yoshida K. Kuwano K. Hagimoto N. Watanabe K. Matsuba T. Fujita M. (2002). MAP Kinase Activation and Apoptosis in Lung Tissues from Patients with Idiopathic Pulmonary Fibrosis. J. Pathol. 198 (3), 388–396. 10.1002/path.1208 12375272

[B39] Zhang Y. Lu P. Qin H. Zhang Y. Sun X. Song X. (2021). Traditional Chinese Medicine Combined with Pulmonary Drug Delivery System and Idiopathic Pulmonary Fibrosis: Rationale and Therapeutic Potential. Biomed. Pharmacother. 133, 111072. 10.1016/j.biopha.2020.111072 33378971 PMC7836923

[B40] Zhu D. W. Yu Q. Sun J. J. Shen Y. H. (2021). Evaluating the Therapeutic Mechanisms of Selected Active Compounds in Houttuynia Cordata Thunb. In Pulmonary Fibrosis via Network Pharmacology Analysis. Front. Pharmacol. 12, 733618. 10.3389/fphar.2021.733618 34658873 PMC8514782

