# Supplementary figures and images for: Exploring the Anti-Pulmonary Fibrosis Mechanism of Jingyin Granule by Network Pharmacology Strategy
Source: Front Pharmacol. 2022 Feb 11;13:825667. doi: 10.3389/fphar.2022.825667 (PMC8874130; doi:10.3389/fphar.2022.825667)

## Slide 1
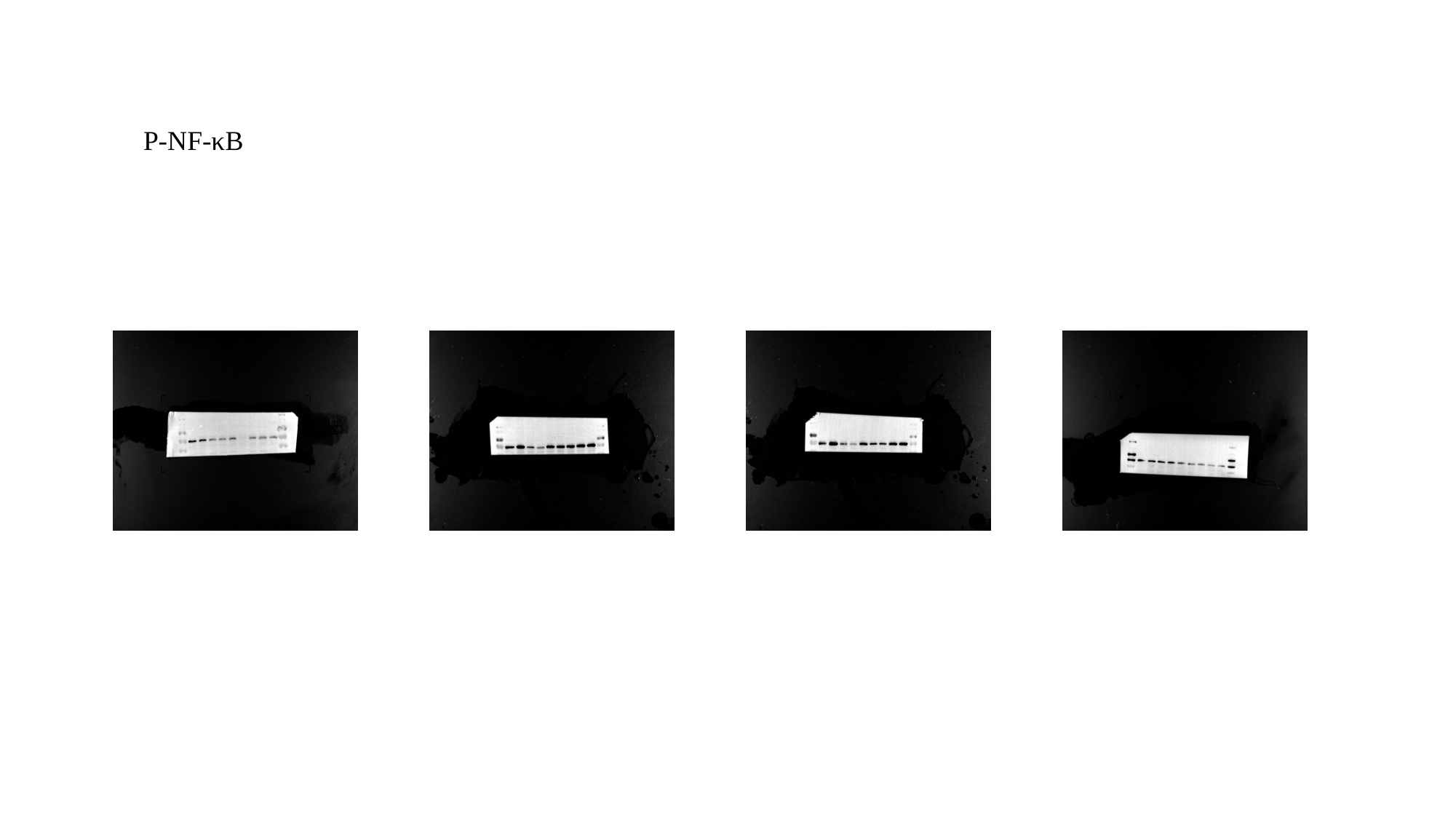

P-NF-κB

## Slide 2
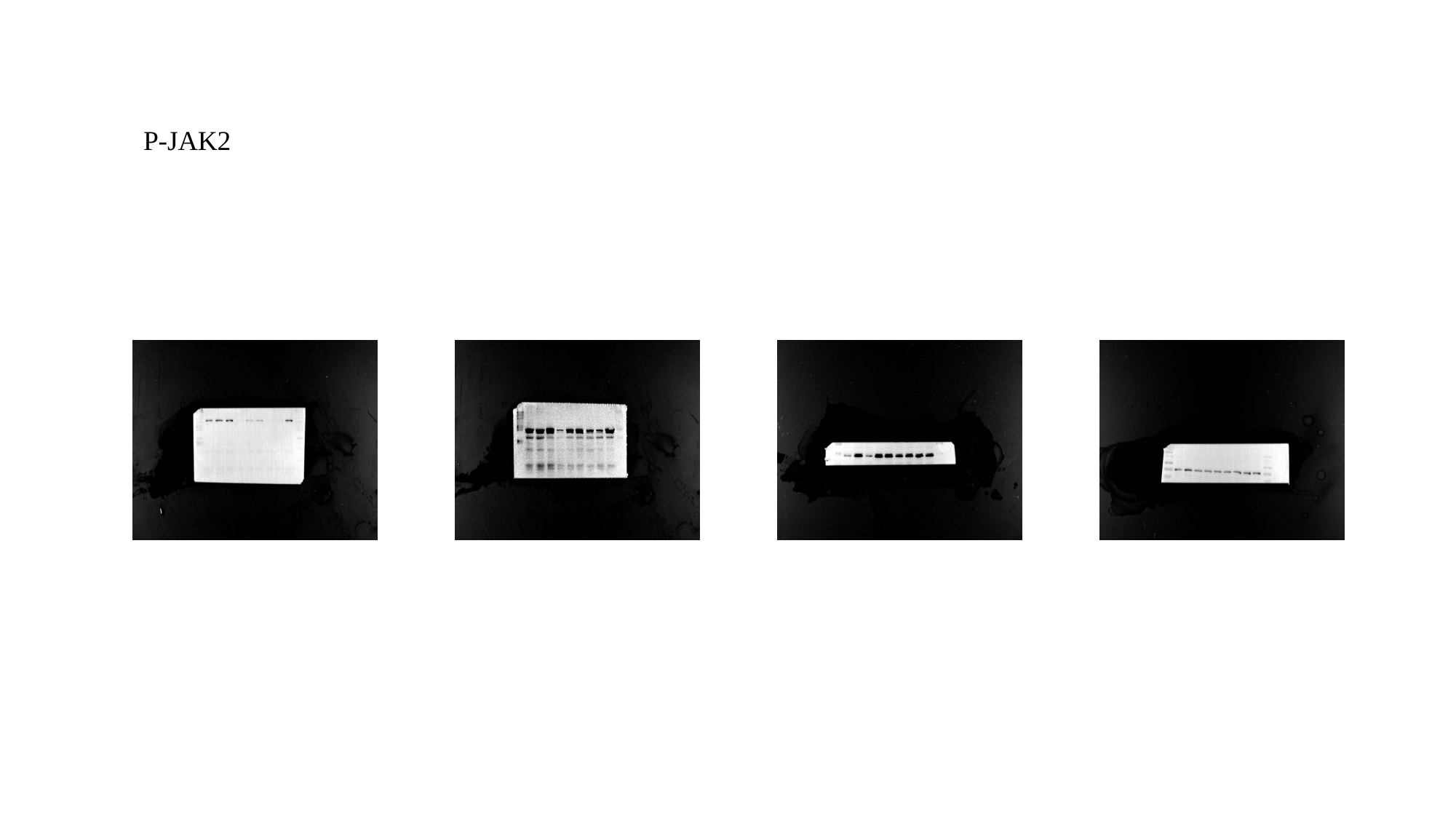

P-JAK2

## Slide 3
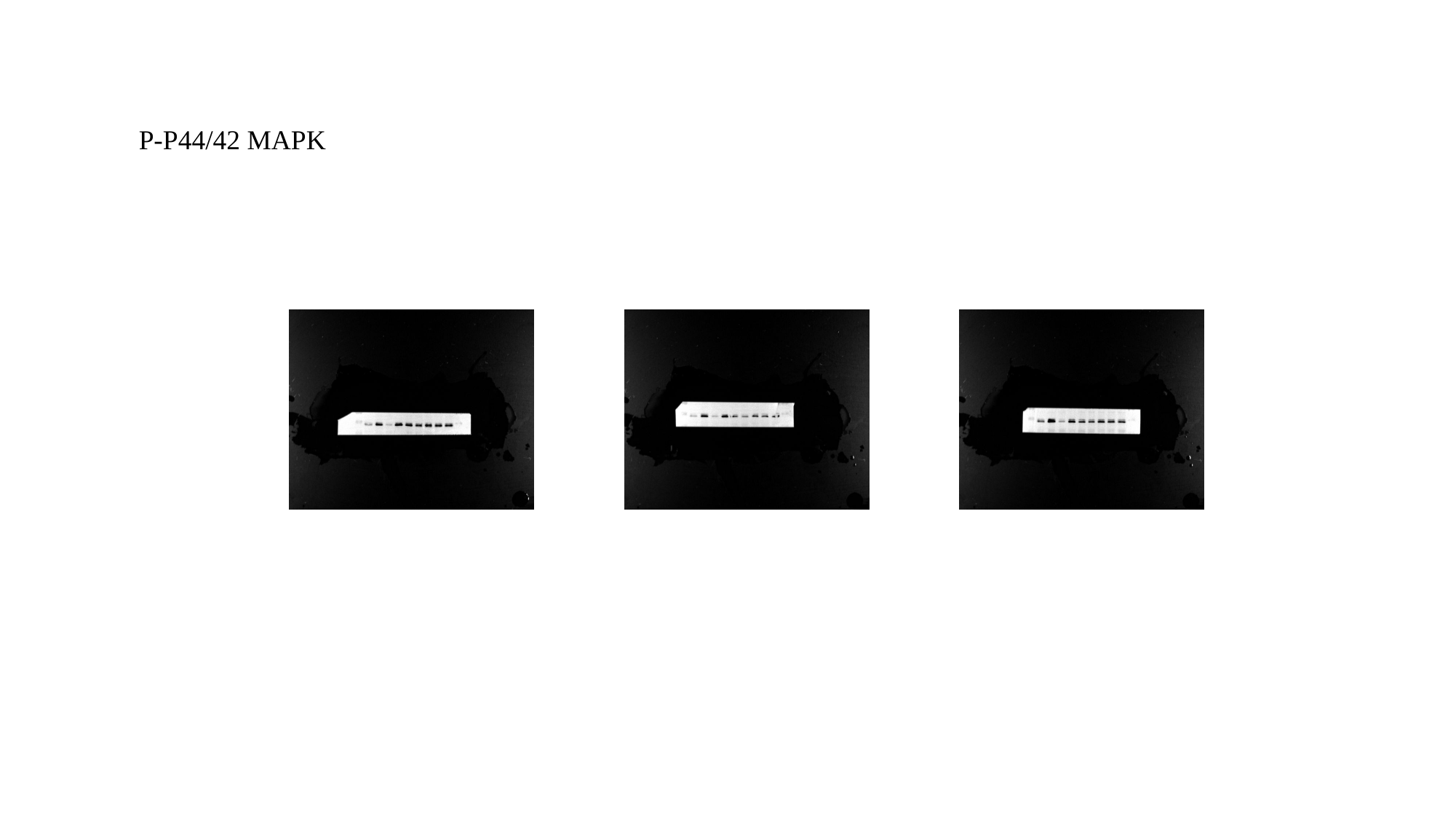

P-P44/42 MAPK

## Slide 4
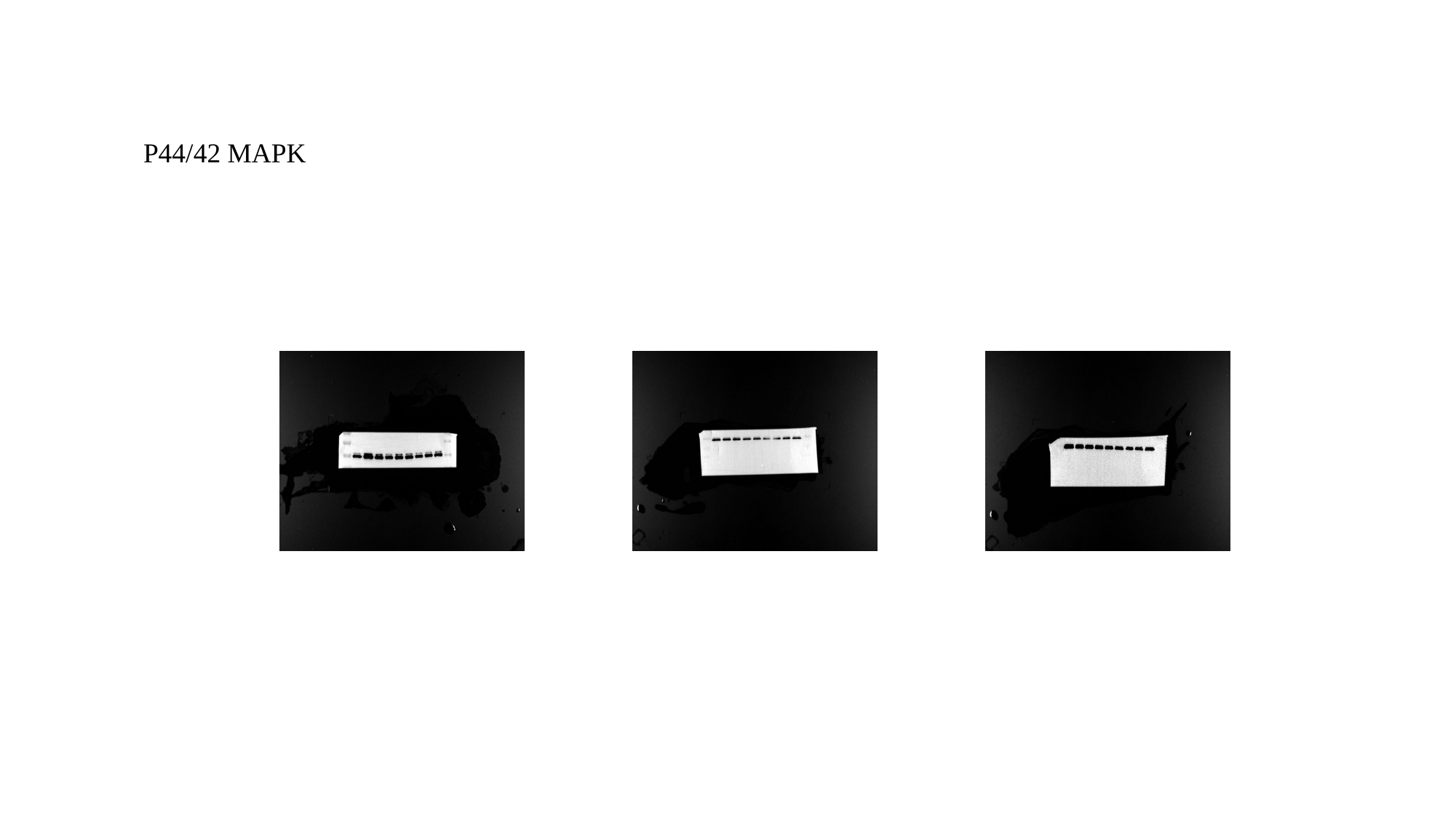

P44/42 MAPK

## Slide 5
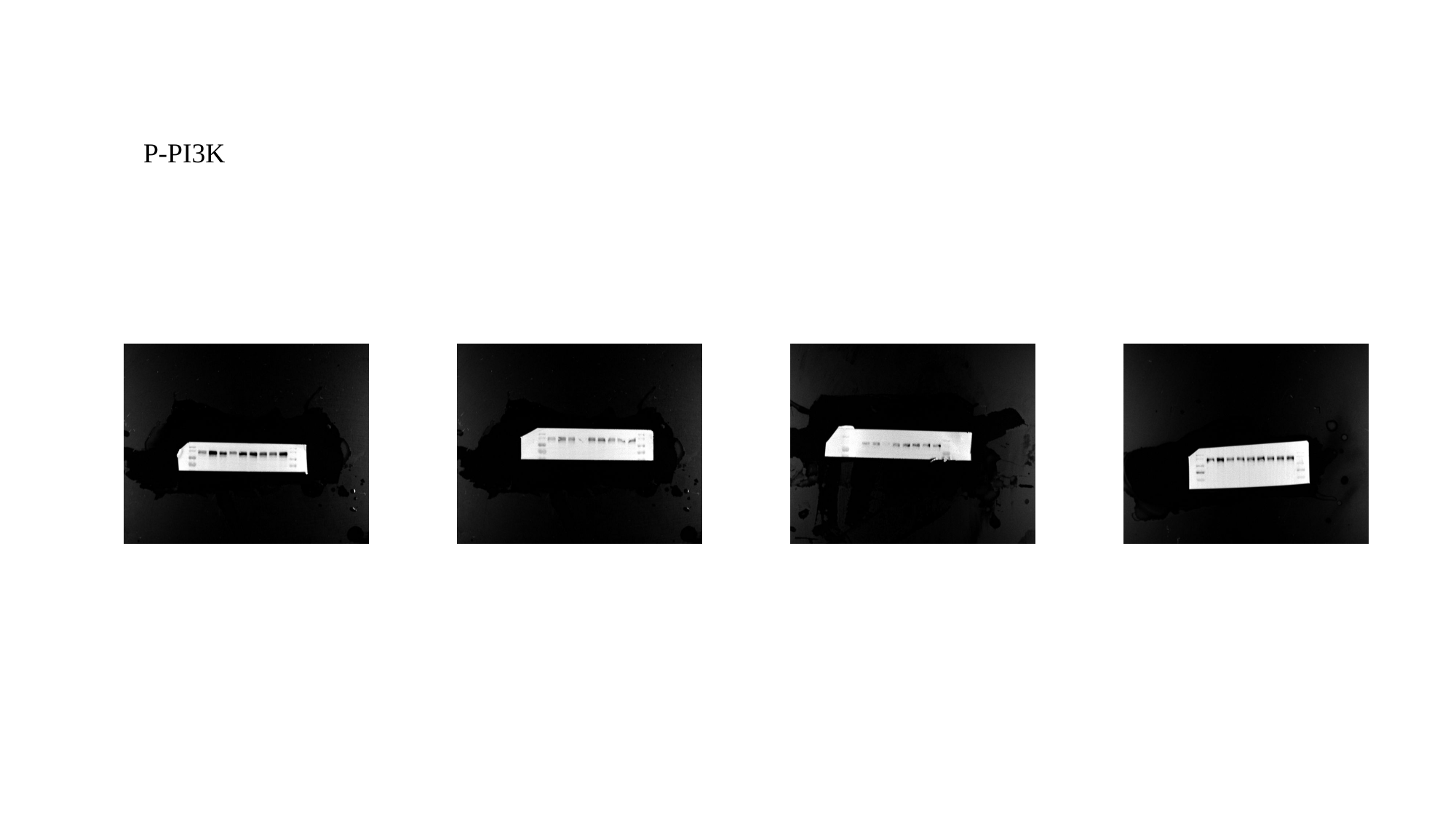

P-PI3K

Supplement: Supplementary file 1 [file Presentation1.PPTX]

## Slide 1
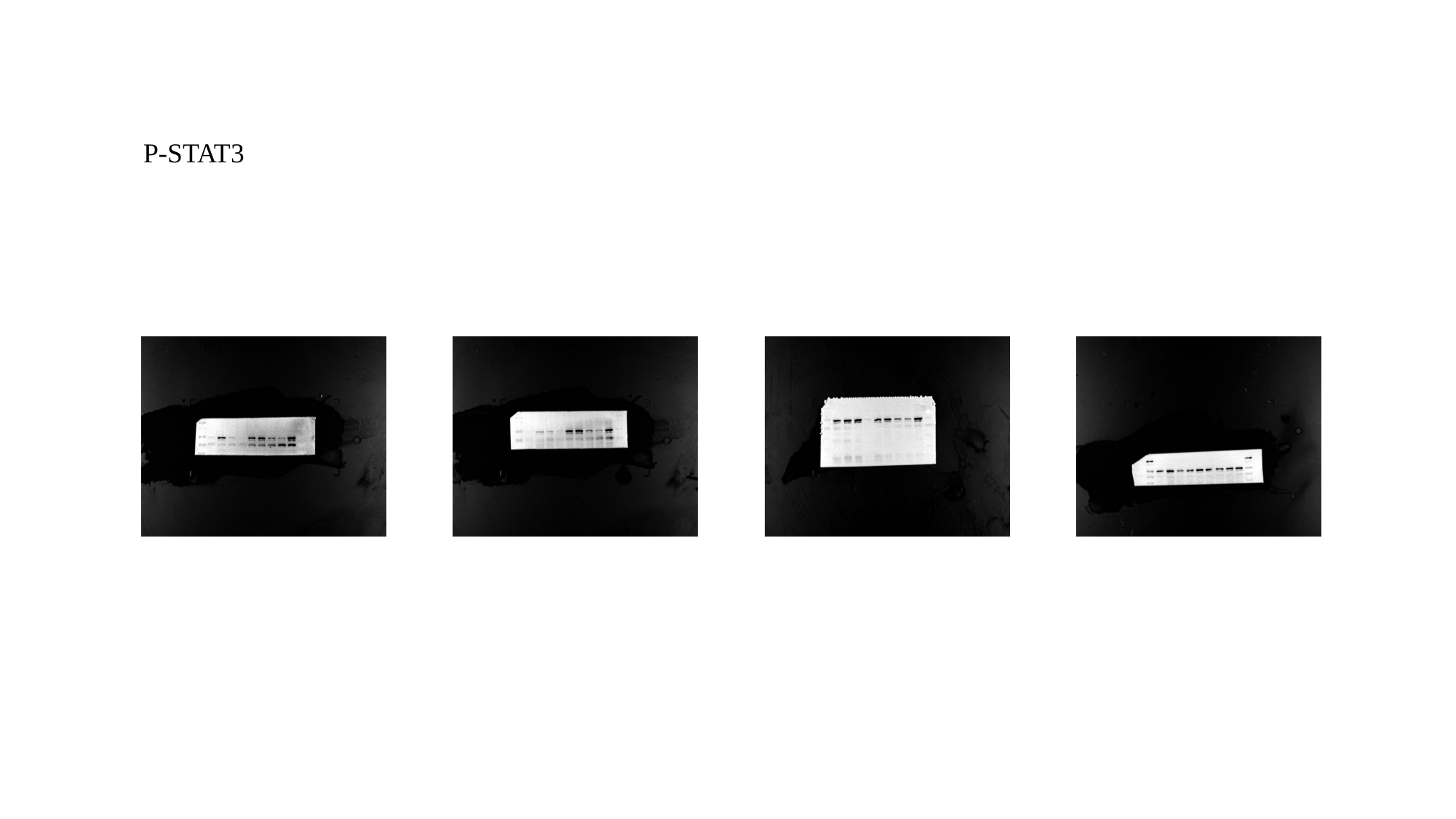

P-STAT3

## Slide 2
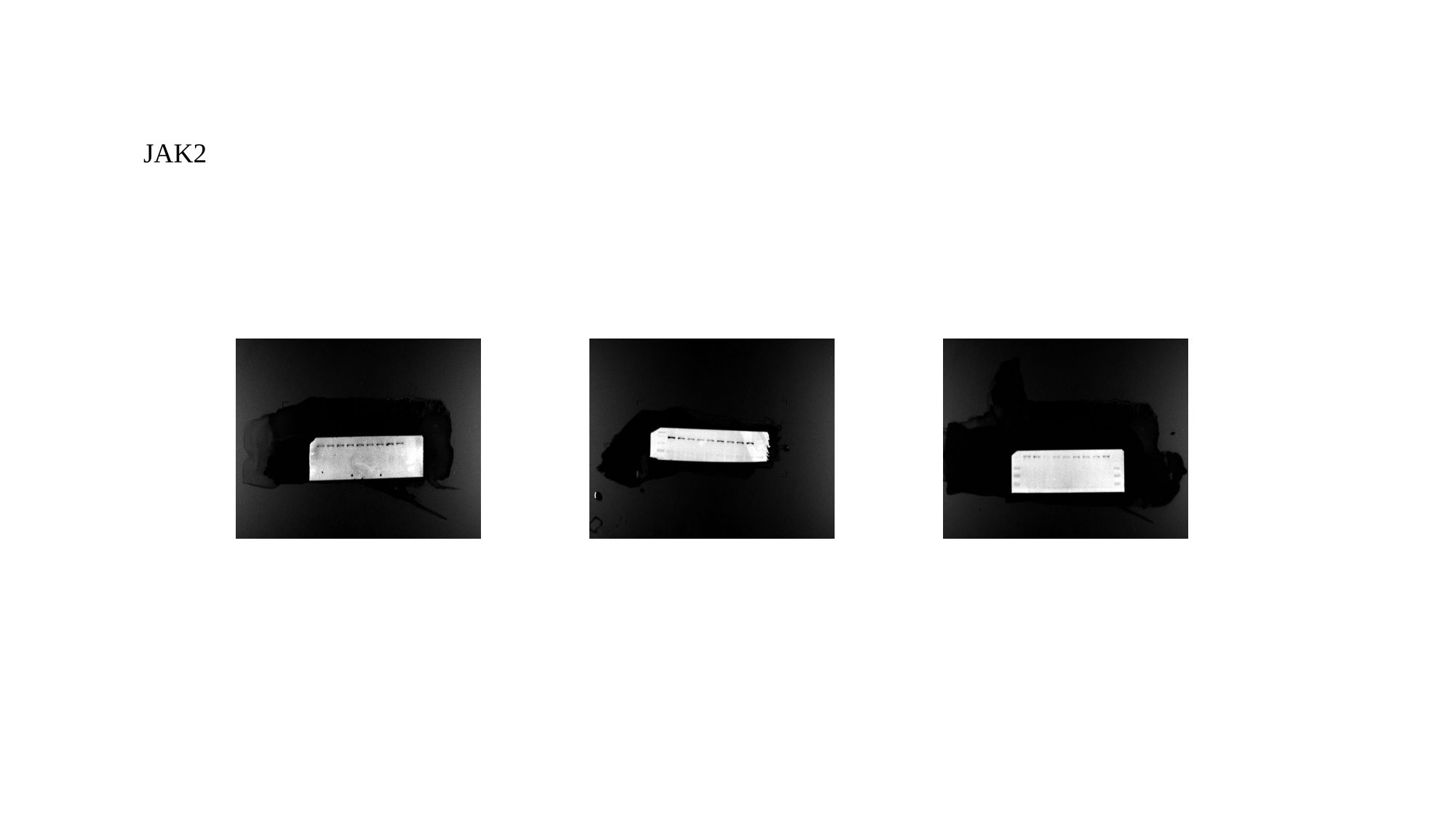

JAK2

## Slide 3
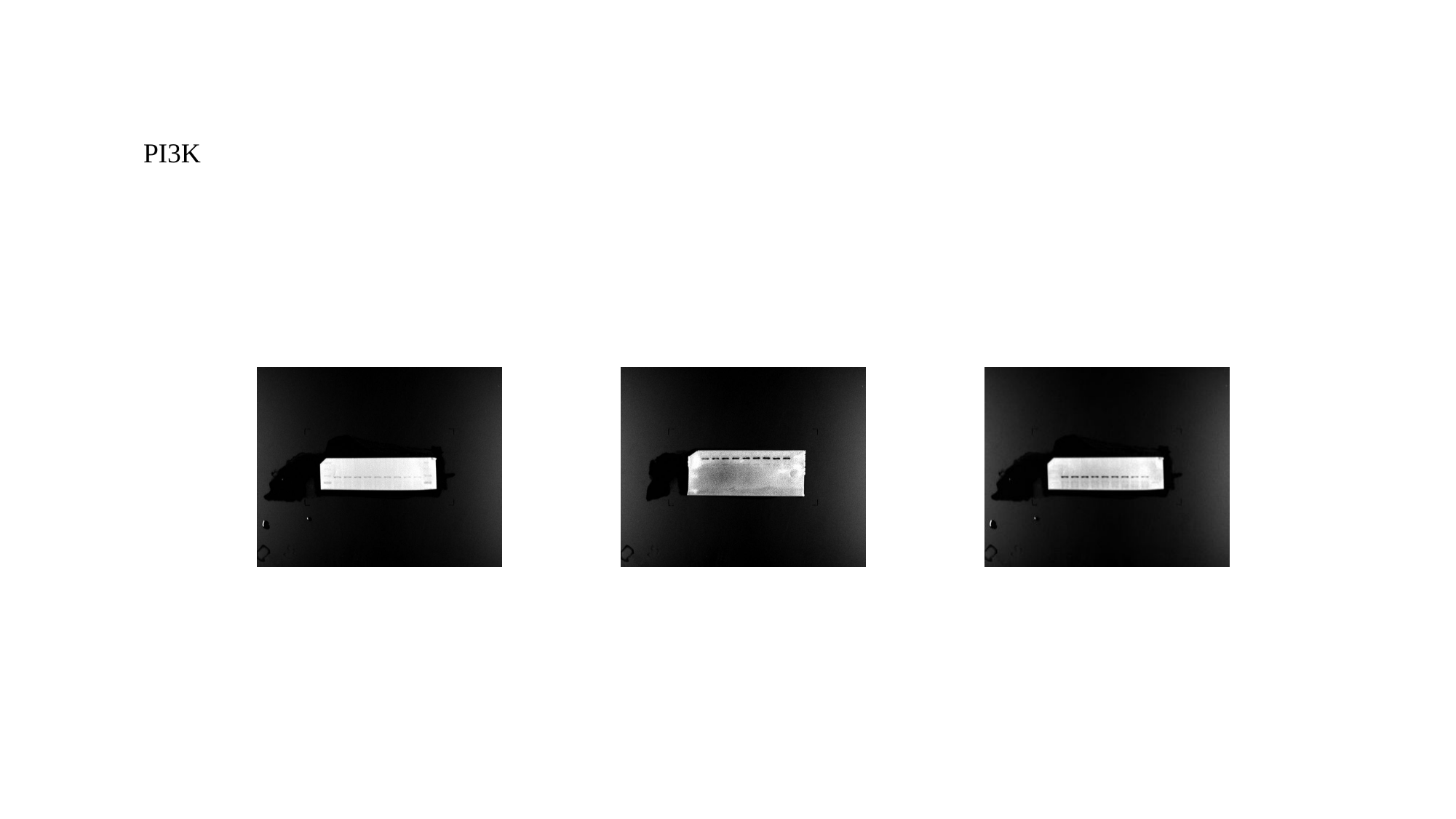

PI3K

## Slide 4
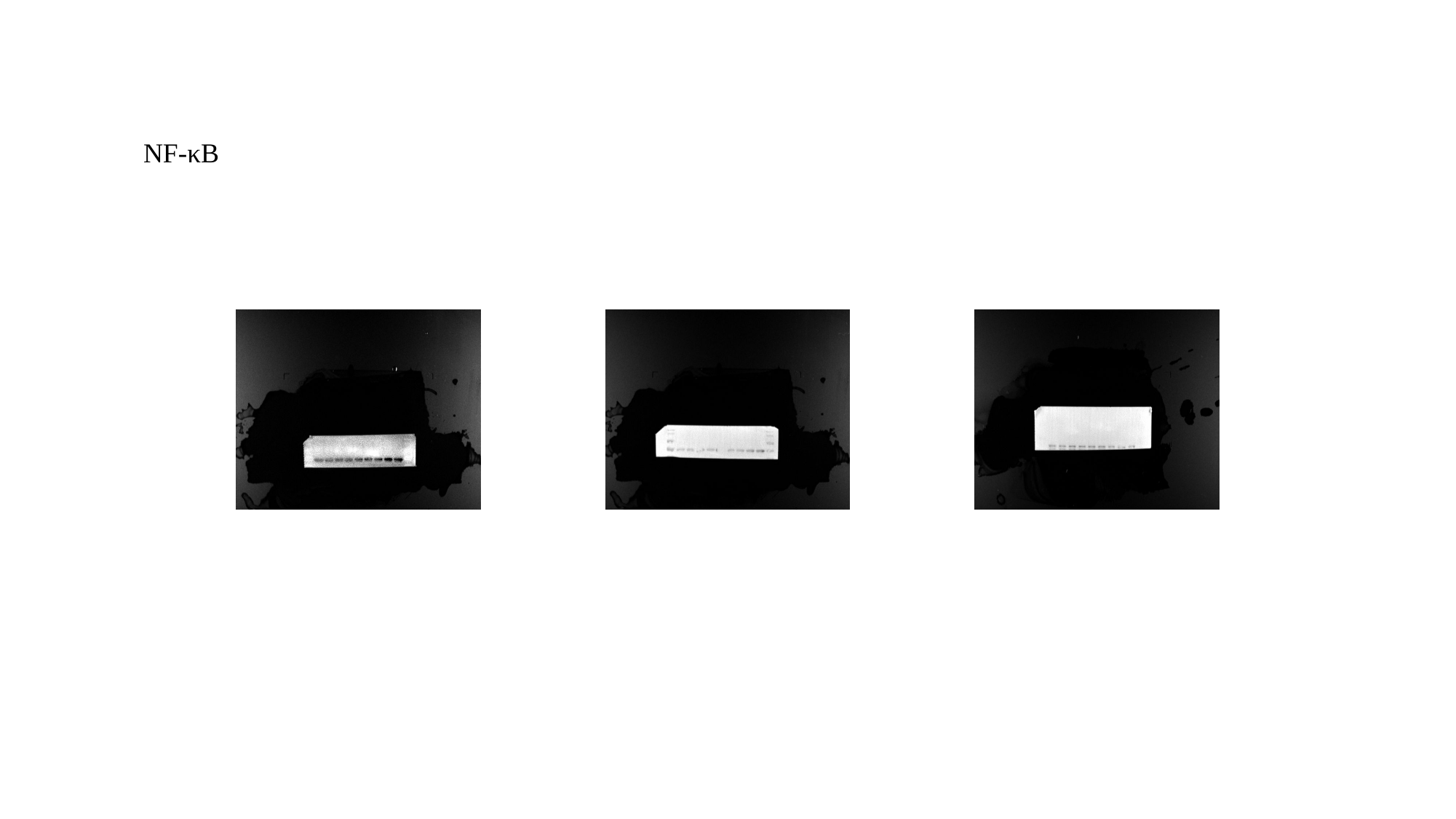

NF-κB

Supplement: Supplementary file 2 [file Presentation3.PPTX]

## Slide 1
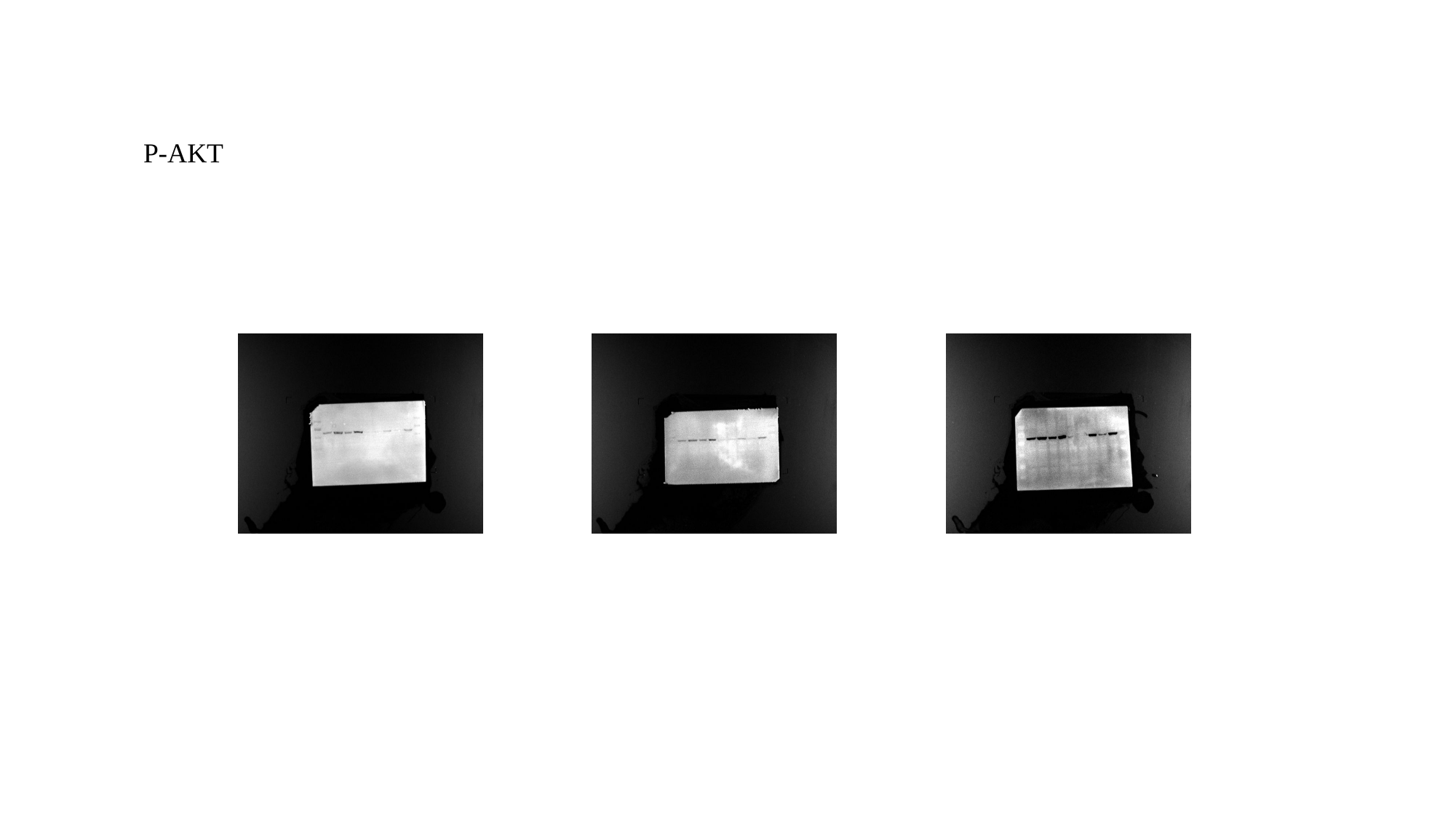

P-AKT

## Slide 2
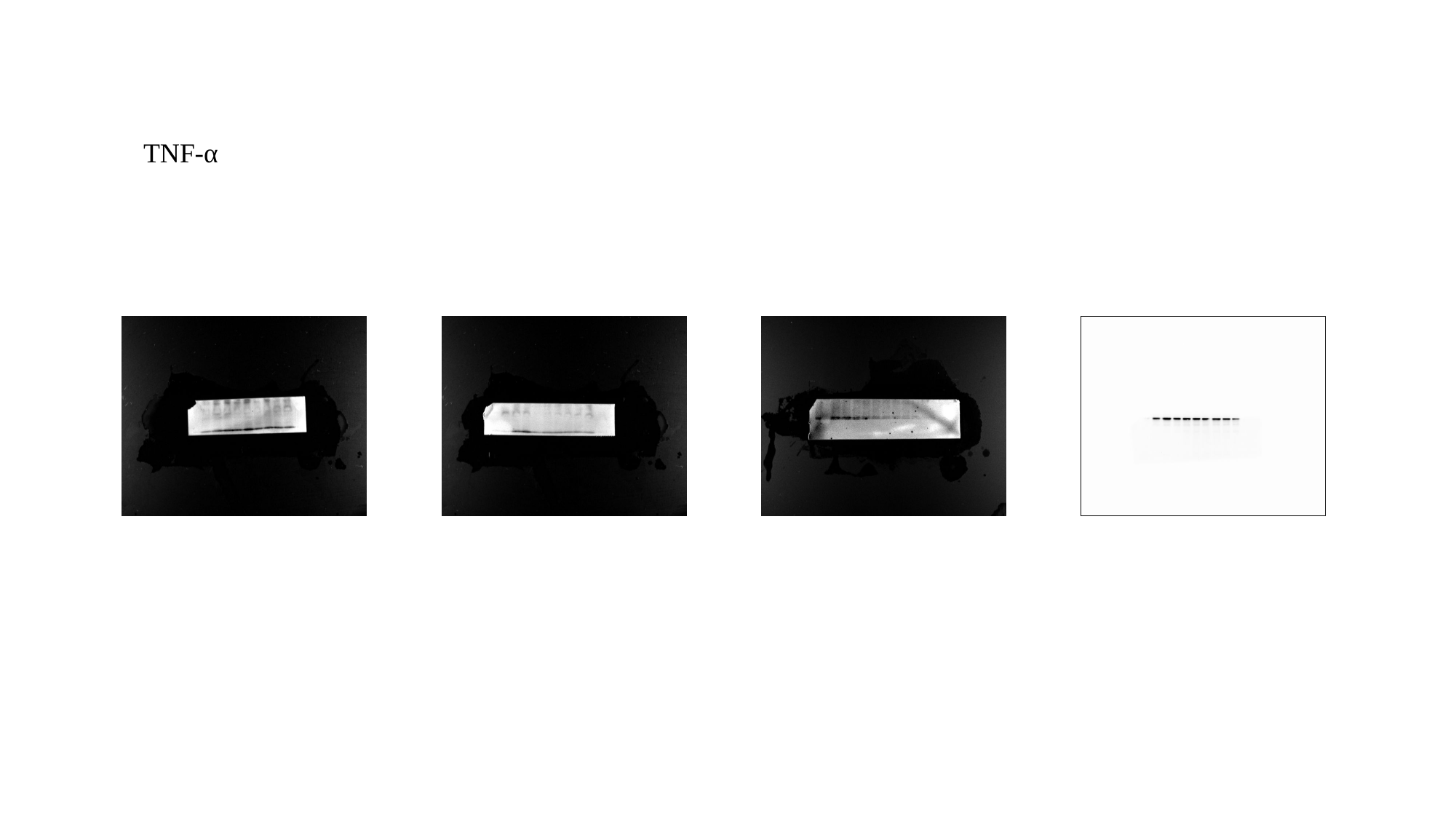

TNF-α

## Slide 3
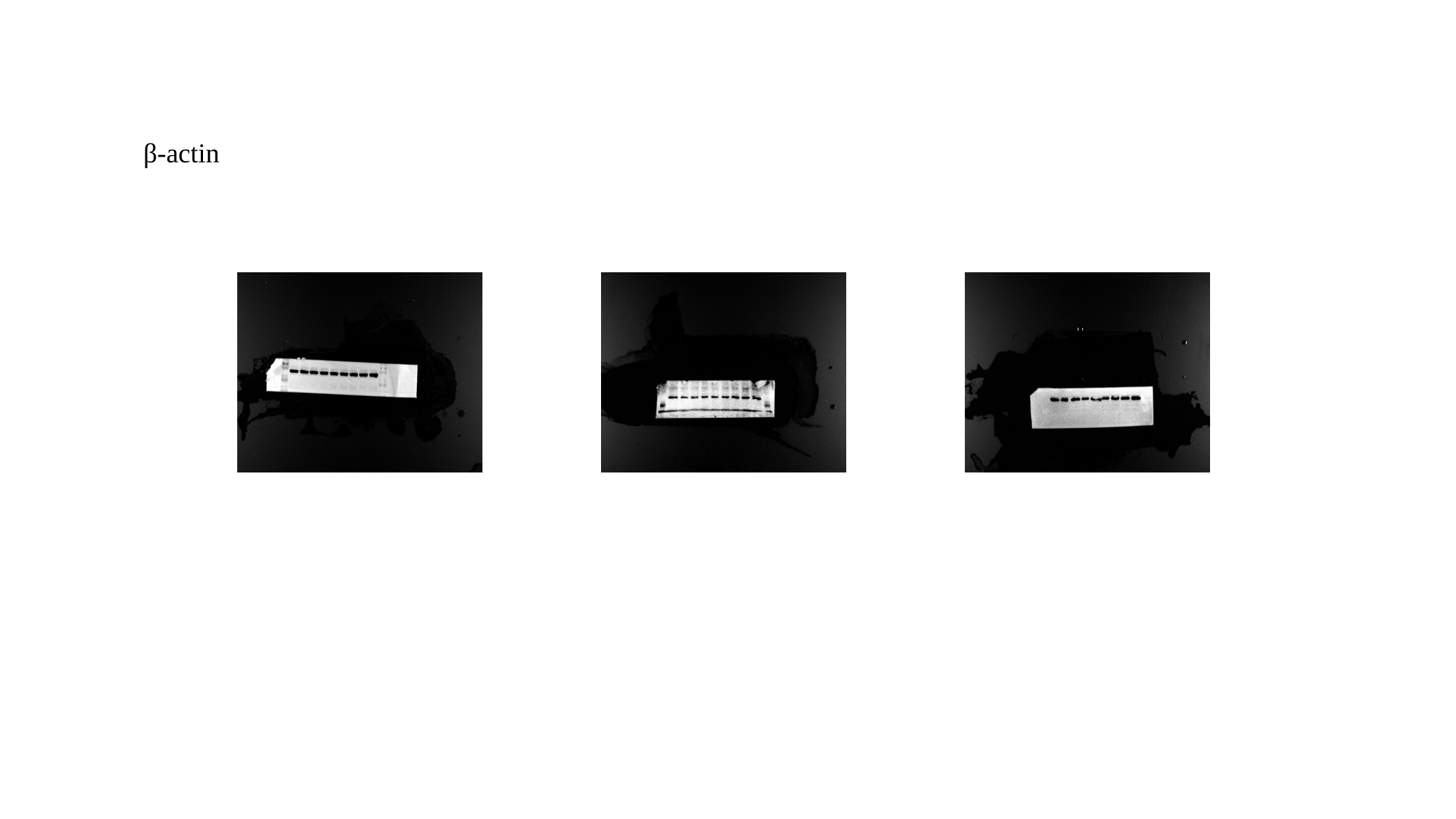

β-actin

## Slide 4
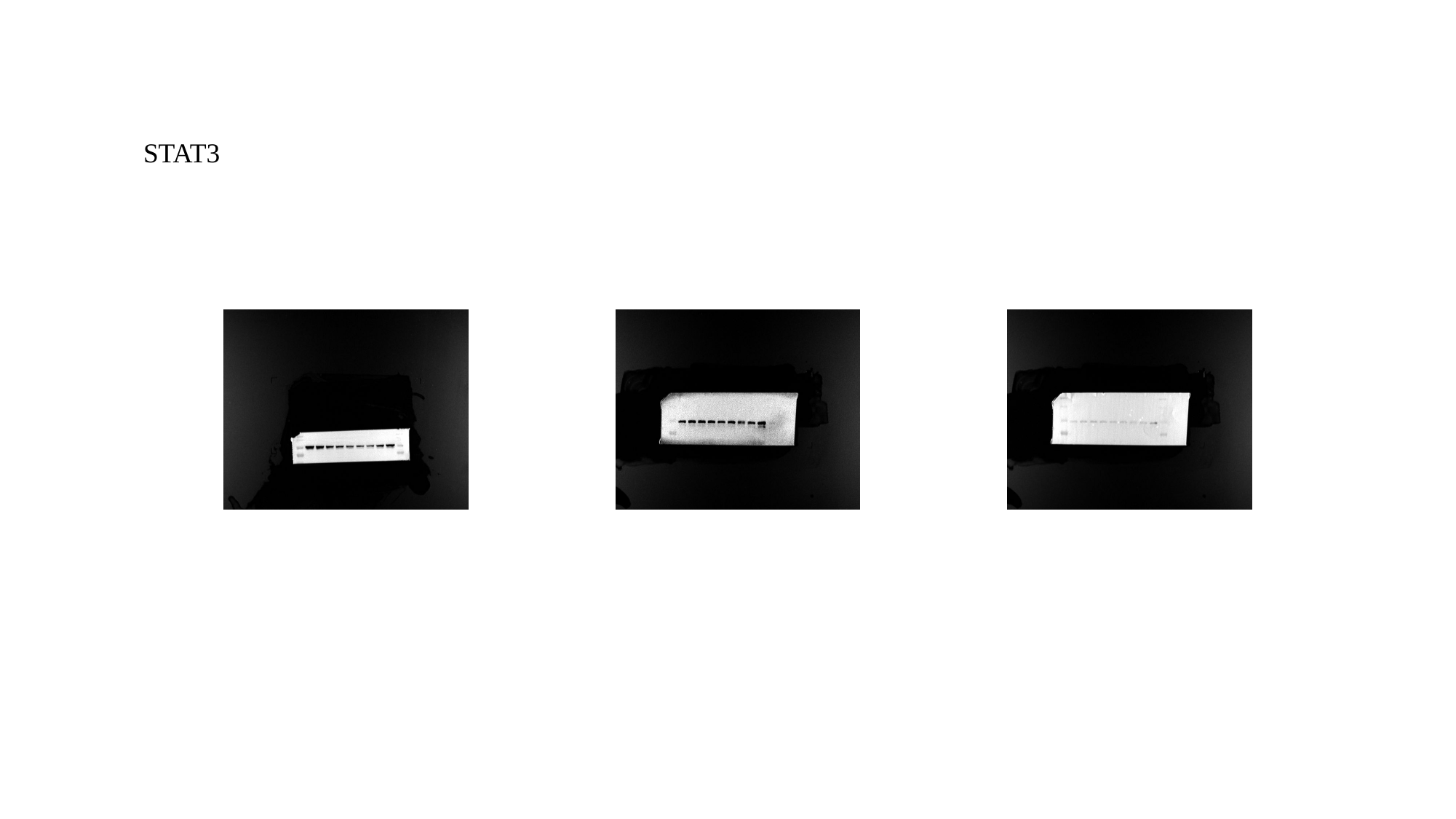

STAT3

## Slide 5
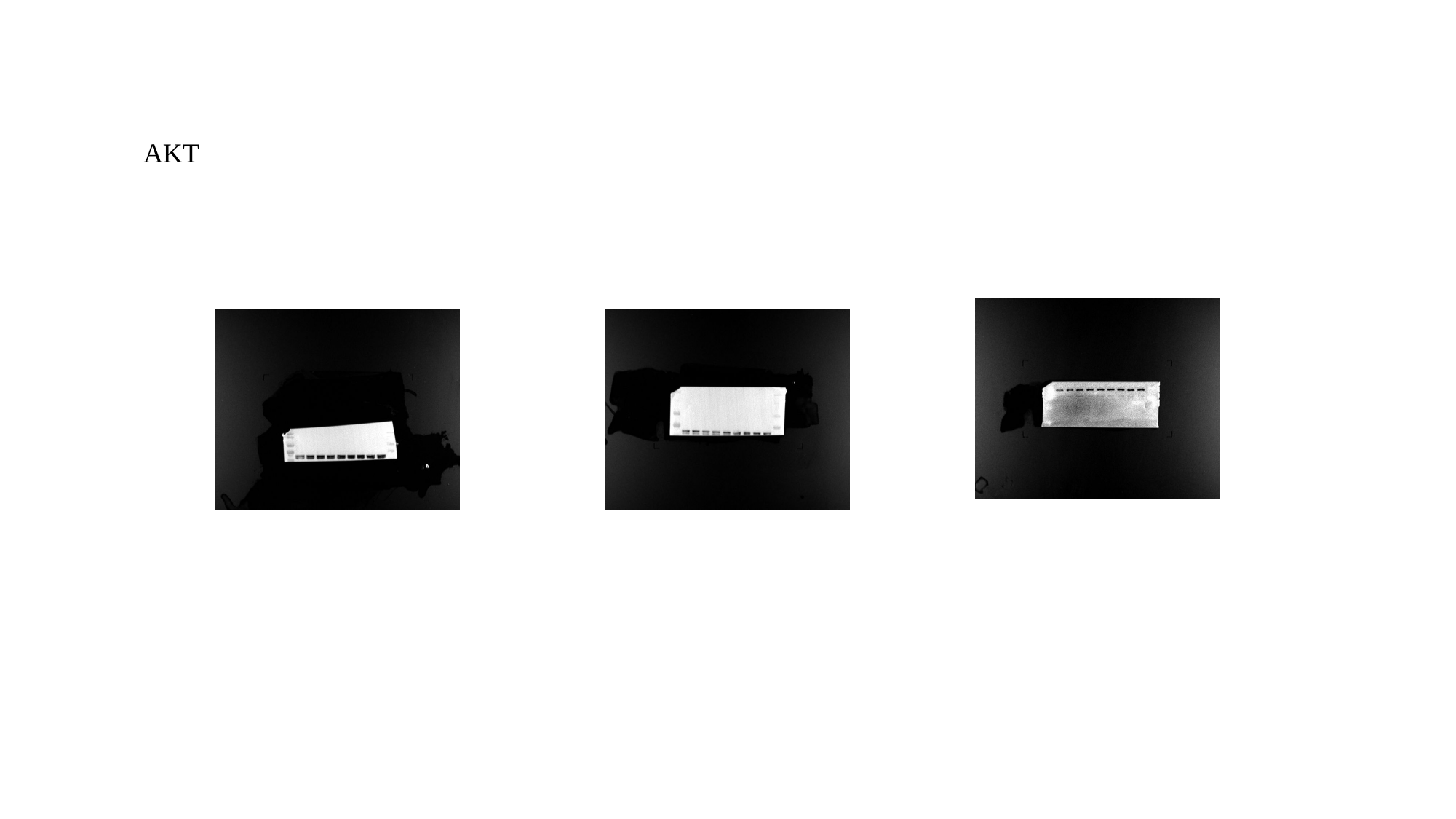

AKT

Supplement: Supplementary file 3 [file Presentation2.PPTX]
